# CO_2_/carbonate-mediated electrochemical water oxidation to hydrogen peroxide

**DOI:** 10.1038/s41467-022-30251-5

**Published:** 2022-05-13

**Authors:** Lei Fan, Xiaowan Bai, Chuan Xia, Xiao Zhang, Xunhua Zhao, Yang Xia, Zhen-Yu Wu, Yingying Lu, Yuanyue Liu, Haotian Wang

**Affiliations:** 1 Department of Chemical and Biomolecular Engineering, Rice University, Houston, TX 77005 USA; 2 State Key Laboratory of Chemical Engineering, Institute of Pharmaceutical Engineering, College of Chemical and Biological Engineering, Zhejiang University, Hangzhou, 310027 China; 3 Texas Materials Institute and Department of Mechanical Engineering, The University of Texas at Austin, Austin, TX 78712 USA; 4 Smalley-Curl Institute, Rice University, Houston, TX 77005 USA; 5 Department of Materials Science and NanoEngineering, Rice University, Houston, TX 77005 USA; 6 Department of Chemistry, Rice University, Houston, TX 77005 USA

**Keywords:** Catalytic mechanisms, Electrocatalysis, Electrocatalysis

## Abstract

Electrochemical water oxidation reaction (WOR) to hydrogen peroxide (H_2_O_2_) via a 2e^−^ pathway provides a sustainable H_2_O_2_ synthetic route, but is challenged by the traditional 4e^−^ counterpart of oxygen evolution. Here we report a CO_2_/carbonate mediation approach to steering the WOR pathway from 4e^−^ to 2e^−^. Using fluorine-doped tin oxide electrode in carbonate solutions, we achieved high H_2_O_2_ selectivity of up to 87%, and delivered unprecedented H_2_O_2_ partial currents of up to 1.3 A cm^−2^, which represents orders of magnitude improvement compared to literature. Molecular dynamics simulations, coupled with electron paramagnetic resonance and isotope labeling experiments, suggested that carbonate mediates the WOR pathway to H_2_O_2_ through the formation of carbonate radical and percarbonate intermediates. The high selectivity, industrial-relevant activity, and good durability open up practical opportunities for delocalized H_2_O_2_ production.

## Abstract (web summary)

Electrochemical H_2_O oxidation to H_2_O_2_ is challenged by the competitive O_2_ evolution reaction. Here, the authors report a CO_2_/carbonate mediation approach to steering the H_2_O oxidation pathway from O_2_ evolution to H_2_O_2_ generation.

## Introduction

Electrochemical water (H_2_O) oxidation to hydrogen peroxide (H_2_O_2_) via a 2e^−^ pathway (2e^−^-WOR) represents a green and sustainable route to produce H_2_O_2_ compared to traditional anthraquinone process, but is currently challenged by low selectivity and activity due to strong competition from the typical 4e^−^ oxygen evolution reaction pathway (OER or 4e^−^-WOR)^1–6^. Traditional approaches to promoting 2e^−^-WOR to H_2_O_2_ have been mostly focused on exploring catalysts with relatively weak binding strength with intermediate O species compared to that in the 4e^−^ counterpart^7–11^. These catalysts (typically made of inert metal oxides^7–10^ as well as other materials^12–14^) requires large overpotentials to activate the water oxidation step, but their 2e^−^-WOR current densities are usually limited at ~10 to 200 mA cm^−2^, as the extra overpotentials to drive larger currents would start to push the water oxidation reaction all the way down to O_2_ with significantly decreased H_2_O_2_ selectivity^7–11,15,16^. As a result, the state-of-the-art 2e^−^-WOR performances are still far below the requirements in practical applications^7,17^.

Reaction redox mediators as “electron shuttles” have been playing important roles in facilitating desired reaction pathways (especially for intermediate products) in electrocatalysis^18,19^, electroorganic synthesis^20–22^ and bioelectrocatalysis^23^, and could become a solution to the H_2_O_2_ activity-selectivity dilemma. While exploring such kind of reaction mediators for selective 2e^−^-WOR is challenging, our nature may have an answer for us. H_2_O_2_ commonly exists in biosystems as one type of reactive oxygen species (ROS)^24^ which are essential in signal transduction^25,26^. However, researchers have found out that high concentrations of CO_2_ could cause ROS burst (rapid release of ROS from cells), leading to destructions of redox-sensitive proteins and cell structures (Supplementary Note 1)^27,28^.

**Fig. 1:**
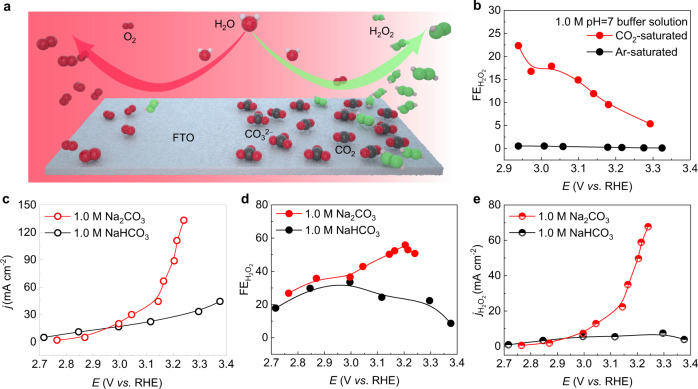
CO_2_/Carbonate promotion effects in 2e^−^-WOR. **a** Schematic illustration of CO_2_/carbonate promoted electrochemical H_2_O oxidation to H_2_O_2_. With the mediator effects of CO_2_/carbonate, the WOR reaction pathway could change from 4e^−^ towards O_2_ to 2e^−^ towards H_2_O_2_. **b** H_2_O_2_ selectivity in Ar-saturated and CO_2_-saturated 1.0 M sodium phosphate buffer solution. CO_2_ serves as a promoter for 2e^−^-WOR, as the H_2_O_2_ selectivity increased by orders of magnitude compared to that without CO_2_. **c**–**e** *I*–*V* curves, H_2_O_2_ FEs, and H_2_O_2_ partial current densities in 1.0 M NaHCO_3_ and 1.0 M Na_2_CO_3_, respectively. The maximum H_2_O_2_ FE in 1.0 M NaHCO_3_ was 34% with a H_2_O_2_ partial current density of 5.6 mA cm^−2^. In comparison, the maximum H_2_O_2_ FE in 1.0 M Na_2_CO_3_ was 56%, and the maximum H_2_O_2_ partial current density was 68 mA cm^−2^.

Inspired by this phenomenon and previous studies in electrogenerated chemiluminescence^29,30^ and peroxycarbonate synthesis^31^, here we hypothesize that CO_2_ (or carbonate as its ionic form in water) may serve as an effective mediator to promote the H_2_O_2_ pathway in electrochemical water oxidation (Fig. 1a)^32–34^. Using fluorine doped tin oxide (FTO) as a model catalyst electrode, we demonstrated high H_2_O_2_ selectivity up to 87%, delivered high H_2_O_2_ partial current densities up to 1.3 A cm^−2^, and achieved a long-term stable and continuous H_2_O_2_ generation for 250 hours with over 80% H_2_O_2_ selectivity at 150 mA cm^−2^ current density in carbonate solutions. The electrochemical performance of our work represents orders of magnitude improvement compared to previous works. We studied the mechanism of the carbonate mediations effects using molecular dynamics simulations, coupled with electron paramagnetic resonance and isotope labeling experiments. Simulation and experimental results suggested that carbonate mediates the WOR pathway through carbonate radical and percarbonate intermediates.

## Results and discussion

### Verification of the CO_2_/carbonate mediation effects

To support the CO_2_/carbonate mediator strategy, an oxidation catalyst electrode which meets the following criteria is a prerequisite: first, it should be an inert catalyst for the 4e^−^ oxygen evolution reaction (OER); second, it has a high electrical conductivity to deliver high currents; third, it should remain stable under high oxidation potentials in water. After an initial screening process, FTO (Supplementary Fig. 1) was selected as the catalytic electrode compare to other materials (Supplementary Fig. 2). While FTO has also been used in some previous studies of photoelectrochemical or electrochemical water oxidation reaction, in most cases it was used as the substrate for studying other catalytic materials^7,8^. Additionally, the H_2_O_2_ selectivity and activity on FTO reported before are rather low^8^, and the surface reaction mechanism of 2e^−^-WOR is still unclear yet. We evaluated the electrochemical WOR performance using a standard three-electrode setup in an H-type cell. Sodium phosphate buffer (0.65 M Na_2_HPO_4_ and 0.35 M NaH_2_PO_4_, pH ~7) was chosen as the aqueous electrolyte, due to its high stability under oxidative potentials^35^ and strong buffering capability^36^, for investigating the possible impacts of CO_2_ on WOR pathways (Fig. 1b and Supplementary Fig. 3). In Ar-saturated 1.0 M sodium phosphate buffer, only trace amount of H_2_O_2_ was detected within a wide range of applied water oxidation potentials, with H_2_O_2_ Faradaic efficiencies (FEs) less than 1% (Fig. 1b). This result suggests that FTO presents an intrinsic selectivity towards the 4e^−^-WOR pathway. Surprisingly, when the solution was saturated with CO_2_, a significant jump of H_2_O_2_ selectivity was achieved under the same reaction conditions (Fig. 1b), supporting our hypothesis that CO_2_ can play a role in steering the WOR reaction pathway towards H_2_O_2_.

**Fig. 2:**
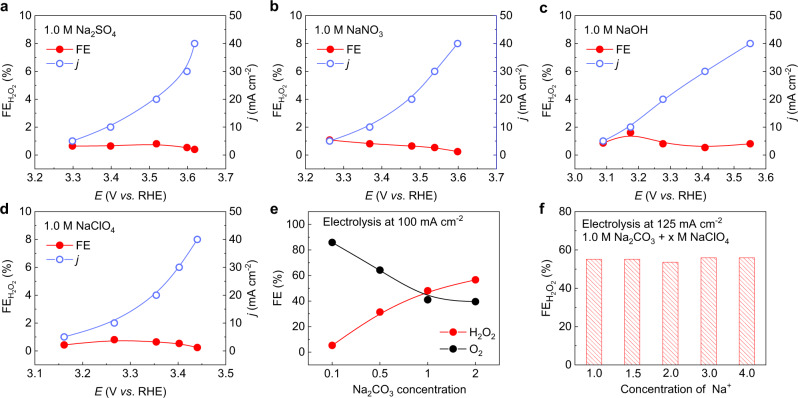
Impacts of anion species and anion/cation concentrations on 2e^−^-WOR. **a**–**d** *I*–*V* curves and H_2_O_2_ FEs of FTO catalyst using 1.0 M Na_2_SO_4_, 1.0 M NaNO_3_, 1.0 M NaOH, and 1.0 M NaClO_4_, respectively. The H_2_O_2_ FEs in these electrolytes were lower than 2%, indicating there are no promotion effects for H_2_O_2_ formation using these anion species. **e** Dependence of H_2_O_2_ and O_2_ selectivity on Na_2_CO_3_ concentration at 100 mA cm^−2^. The H_2_O_2_ FE was increased with increased Na_2_CO_3_ concentration, while the FE of side product O_2_ was correspondingly decreased, indicating that Na_2_CO_3_ could be directly involved in the 2e^−^-WOR process. **f** H_2_O_2_ FE in 1.0 M Na_2_CO_3_ with different concentrations of NaClO_4_.

As CO_2_ exists in different types of species in aqueous solutions^37^, including dissolved CO_2_, carbonate, and bicarbonate, we therefore designed control experiments to better identify the key factors that are at play in promoting H_2_O_2_ generation. We first used 1.0 M NaHCO_3_ as the electrolyte, where bicarbonate is the dominant species, to evaluate FTO’s 2e^−^-WOR performance. As shown in Fig. 1c–e and Supplementary Fig. 4, the peak H_2_O_2_ FE was ~ 34%, corresponding to a H_2_O_2_ partial current density of only 5.6 mA cm^−2^. We further switched the electrolyte to carbonate dominated 1.0 M Na_2_CO_3_ solution and performed the same test, and observed a drastic change. The H_2_O_2_ FE jumped up to a maximal of 56% at 3.2 V versus reversible hydrogen electrode (*vs*. RHE) with a significantly improved H_2_O_2_ partial current density of over 50 mA cm^−2^ (Fig. 1e), representing over one order of magnitude increase compared to that in either CO_2_ saturated electrolyte or bicarbonate electrolyte. To explore if this promotion effect on 2e^−^-WOR pathway is an intrinsic catalytic property of the FTO catalyst, or specifically exists in the CO_2_/bicarbonate/carbonate systems, we also tested the H_2_O_2_ selectivity in other commonly used electrolytes containing different anion species, including sodium sulfate, sodium nitrate, sodium hydroxide, sodium perchloride, as well as the sodium phosphate buffer we showed earlier (Fig. 2a–d and Supplementary Fig. 5). To further confirm the carbonate promotion effects, we then tested the electrochemical water oxidation performance in Na_2_CO_3_ + NaOH electrolyte (Supplementary Fig. 6a). In alkaline electrolytes, OH^−^ absorption plays a key role in oxygen evolution reaction^38^. With the increase of NaOH concentration, OH^−^ absorption will decrease the coverage ratio of carbonate absorption on FTO surface, and thus leading to decreased H_2_O_2_ selectivity and increased OER, which further demonstrates that high pH is not the reason for high H_2_O_2_ selectivity when compared to bicarbonate solutions. As a result, none of them presented any preferences for 2e^−^-WOR pathway, with negligible H_2_O_2_ selectivity of less than 2%, which indicates the participation of CO_2_-related species in the electrochemical H_2_O-to-H_2_O_2_ conversion process on the FTO surface.

Since in carbonate solution the FTO catalyst exhibited the best H_2_O_2_ generation performance, we thus tested the dependence of H_2_O_2_ selectivity over Na_2_CO_3_ concentration to further reveal the promotion effects of carbonate on 2e^−^-WOR. As shown in Fig. 2e, the H_2_O_2_ FE under 100 mA cm^−2^ current presented a monotonic enhancement from 5% to 56% with increased Na_2_CO_3_ concentration from 0.1 M to 2.0 M, respectively, indicating the critical role of carbonate ions in promoting H_2_O_2_ selectivity. Possible promotion effects from sodium ions were excluded as the H_2_O_2_ FEs were similar under different Na^+^ concentrations (Fig. 2 f). We also examined the O_2_ FEs from the 4e^−^-WOR pathway using gas chromatography quantification, which added together with H_2_O_2_ are close to 100%, suggesting no significant side reactions in this electrochemical system (Fig. 2e, see Methods). In addition, FTO electrodes with different thicknesses, fluorine doping levels, or surface resistivity exhibited quite similar H_2_O_2_ selectivity (Supplementary Fig. 6b), further confirming that the dominant factor on WOR pathway is from carbonate concentrations. Other types of common conducting metal oxides, including alumina-doped zinc oxide and indium tin oxide, were also evaluated in carbonate electrolyte but showed poor stability under the WOR conditions (Supplementary Fig. 2). Titanium mesh, which is an OER inert metal catalyst, also exhibited good 2e^−^-WOR performance in 1.0 M Na_2_CO_3_ with a maximum H_2_O_2_ FE of ~ 47% at 2.36 V (Supplementary Fig. 2e, f). However, it presented poor stability due to possible surface passivation under high oxidation potentials (Supplementary Fig. 2 g). These above experimental results strongly support our hypothesis that CO_2_/carbonate may directly participate in and promote the H_2_O_2_ generation process as a promising 2e^−^-WOR mediator.

### Electrochemical H_2_O_2_ generation at industrial-relevant current densities

**Fig. 3:**
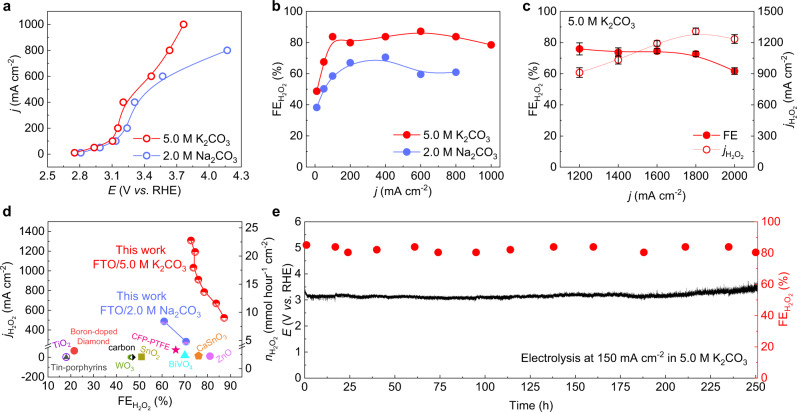
Electrochemical 2e^−^-WOR performance using high concentration carbonate mediator. **a** *I*–*V* curves of FTO catalyst in 2.0 M Na_2_CO_3_ and 5.0 M K_2_CO_3_. K_2_CO_3_ was chosen due to its higher solubility than Na_2_CO_3_. The maximal current density in our standard three-electrode cell was cut at 1 A cm^−2^. **b** The corresponding H_2_O_2_ FEs. The highest FE reached 87% in 5.0 M K_2_CO_3_ with a current density of 600 mA cm^−2^ under 3.46 V. **c** H_2_O_2_ FEs and partial currents in 5.0 M K_2_CO_3_ solution with current densities greater than 1 A cm^−2^. The error bars represent two independent tests. **d** Comparison of H_2_O_2_ selectivity and activity between this work and previous reports^7,9,11–14,41^. **e** The stability test of FTO catalyst by maintaining a 150 mA cm^−2^ WOR current in 5.0 M K_2_CO_3_ solution. Its H_2_O_2_ selectivity and potential remained stable for a 250-hour continuous operation.

To further amplify the carbonate mediation effect for improved H_2_O_2_ generation performance, we evaluated the electrochemical 2e^−^-WOR performance of FTO electrode in high concentration carbonate solutions. Figure 3a, b and Supplementary Fig. 7 show the *I*–*V* curves and corresponding H_2_O_2_ FEs under different potentials. In 2.0 M Na_2_CO_3_ solution, we achieved high H_2_O_2_ FEs of ~60 to 70%, while delivering large current densities of up to 800 mA cm^−2^. This impressive H_2_O_2_ performance can be even further improved by using 5.0 M of K_2_CO_3_. The reason why we chose to use K_2_CO_3_ is due to its higher solubility in water (112.3 g in 100 g water at 25 °C) than that of Na_2_CO_3_ (29.4 g in 100 g water at 25 °C)^39^. The FTO catalyst achieved a 10 mA cm^−2^ onset current density at 2.75 V *vs*. RHE in 5.0 M K_2_CO_3_, which is 50 mV lower than that in 2.0 M Na_2_CO_3_. With the overpotentials gradually increased, the H_2_O_2_ FE quickly ramped up to a plateau of over 80% under a wide range of current densities of up to 1 A cm^−2^ (Fig. 3b). We achieved a maximal H_2_O_2_ FE of 87% at 600 mA cm^−2^, representing a 522 mA cm^−2^ H_2_O_2_ partial current density (Fig. 3b). More impressively, at 3.7 V, the catalyst reached a current density of 1 A cm^−2^ while still maintaining a high H_2_O_2_ selectivity of 78%, achieving an industrial-relevant H_2_O_2_ partial current density of 780 mA cm^−2^. The H_2_O_2_ generation rate can be further boosted to a maximal partial current of 1.3 A cm^−2^ (73% FE at 1.8 A cm^−2^ overall current) using an extended-range power supply. This corresponds to an unprecedented H_2_O_2_ production rate of 24.3 mmol cm^−2^ h^−1^ and is orders of magnitude higher compared to previous reports (Fig. 3c, d). Please be noted here that this power supply was not equipped with a three-electrode system therefore the anode potential could not be accurately measured (see Methods). Such high electrochemical H_2_O_2_ generation rates benefit from the sufficient mass diffusions in aqueous solutions where no triple-phase boundary is needed, which shows an advantage compared to the cathodic H_2_O_2_ generation from 2e^−^-ORR (oxygen reduction reaction) where O_2_ gas diffusions typically limit its reaction rates to hundreds of milliamps per square centimeter (Supplementary Note 2)^2,40^. Finally, long-stability is usually a big challenge for 2e^−^-WOR due to the high oxidation potentials that could damage the electrode^9,11,16,17,41,42^ (Supplementary Table 1). Besides, in traditional batch reactors, as the products continuous to accumulate during the long-term stability test (Supplementary Fig. 8a), the electrolyte environment will be changed and thus the electrocatalytic performance continuously decayed. Our FTO catalyst electrode, coupling with a flow reactor (Supplementary Fig. 8b), presented excellent durability under water oxidation conditions, maintaining a stable potential and high H_2_O_2_ FEs of over 80% to deliver a 150 mA cm^−2^ current for 250 hours (Fig. 3e and Supplementary Table 1).

The good selectivity, activity, and stability of our carbonate mediated 2e^−^-WOR makes it possible for this anode reaction to be coupled with 2e^−^-ORR cathodic reaction to double the efficiency of electrons in producing H_2_O_2_ from both electrodes (Supplementary Fig. 9a). On the anode side, H_2_O can be oxidized to H_2_O_2_ via carbonate mediation by our high-performance 2e^−^-WOR catalyst. On the cathode side, we used oxidized carbon black (demonstrated in our previous study^40^) as the selective 2e^−^-ORR catalyst to reduce O_2_ into H_2_O_2_ (Supplementary Fig. 9b, c)^40,43^. Based on the previous definition of H_2_O_2_ FE on one side of the electrode, the maximal overall H_2_O_2_ FE in this two-electrode system is 200%. As shown in Supplementary Fig. 9d, our system delivered a 100-mA cell current (1 cm^2^ FTO electrode) at 2.5 V cell voltage with a high overall H_2_O_2_ FE of 140%, suggesting a significant improvement compared to either 2e^−^-WOR or 2e^−^-ORR system. Furthermore, to fully use generated H_2_O_2_ and Na_2_CO_3_ mediator, we designed a process (Supplementary Fig. 9a) for a continuous generation of an adduct product between Na_2_CO_3_ and H_2_O_2_ (Na_2_CO_3_ ∙ 1.5H_2_O_2_, Supplementary Fig. 9e–g)^11,44^.

### Mechanism studies

**Fig. 4:**
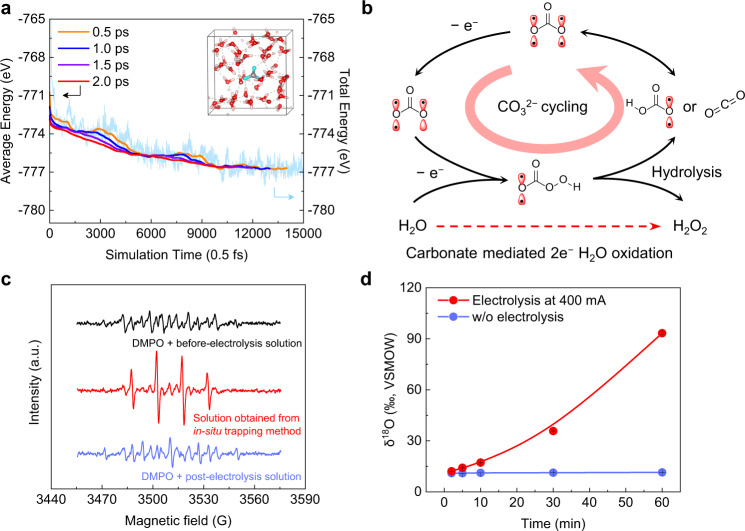
Molecular understanding of the reaction mechanism and experimental detection of key intermediates. **a** Convergence of average energy of CO_3_^•−^ with 50 H_2_O molecules using AIMD. Evaluating average of total energy was done over a varying length of averaging time, from 0.5 ps to 2.0 ps. The time coordinate for the average energy corresponds to the point from which the averaging window begins (e.g., for an averaging window of 0.5 ps, the average that is shown at 500 fs corresponds to the average from 500 fs to 1,000 fs). Cyan, red, light pink, and gray balls represent the O in CO_3_^•−^_,_ the O in H_2_O, H, and C atoms, respectively. **b** Reaction mechanism of carbonate-mediated 2e^−^-WOR to H_2_O_2_. Two possible key intermediates, CO_3_^•−^ and HCO_4_^−^, were proposed, to facilitate the 2e^−^ pathway. **c** EPR spectra of Na_2_CO_3_ electrolyte containing DMPO spin trap. Compared to the solutions of non-electrolysis or post-electrolysis, the solution obtained from the in-situ trapping method under electrolysis exhibit a clear four-line 1:2:2:1 splitting pattern characteristic of the DMPO^•^ − OH adduct, indicating the formation of carbonate radical intermediates under electrolysis. **d** ^18^O isotope abundance in dried carbonate (Methods) at varied isotope exchange time between Na_2_CO_3_ solute and deionized water without or without electrolysis. The ^18^O isotope abundance in dried carbonate under WOR conditions was orders of magnitude higher than that of natural exchange background, indicating the C-O bonding dissociation and formation due to the percarbonate hydrolysis step. The error bars represent two independent tests.

To gain a molecular level understanding of the reaction mechanism in our CO_2_/carbonate mediated 2e^−^-WOR, we employed molecular dynamics simulations, coupled with experimental studies, to reveal the most possible reaction pathway. Reaction intermediates that could exist in electrolyte under the applied high oxidation potentials (~ 3 V vs. RHE)^45^, including CO_3_^•−^, OH^•^, and percarbonates (HCO_4_^−^ or C_2_O_6_^2−^), were taken into consideration^34,46^. We first proposed several possible reaction pathways as summarized in Supplementary Fig. 10. After an initial screening based on theoretical studies and experimental observations, we proposed that the carbonate-mediated water oxidation to H_2_O_2_ could proceed via the following four reaction intermediate steps with most favorable thermodynamics^7,28,47,48^ CO_3_^•−^, HCO_4_^−^, HCO_3_^− ^+ H_2_O_2_, and CO_2_ + H_2_O_2_ + OH^−^. We used ab initio molecular dynamics (AIMD) to evaluate the thermodynamics of these intermediate steps. A 12 Å × 12 Å × 12 Å cubic supercell with 57 water molecules was used to simulate bulk water and maintain a density of 1 g cm^−3^ (Supplementary Fig.11a, b). To assess the energies of these intermediates in ionic form in aqueous solution, seven H_2_O molecules were replaced with CO_3_^•−^ or HCO_4_^−^ in succession (Fig. 4a and Supplementary Fig. 11b, c), while HCO_3_^− ^+ H_2_O_2_ or CO_2_ + H_2_O_2_ + OH^−^ replaced eight H_2_O molecules (Supplementary Fig. 11d, e). Taking the simulation of CO_3_^•−^ as an example here, we first evaluated the convergence of the average energy. Figure 4a showed running average of total energy in different averaging time windows from 0.5 ps to 2.0 ps. We found that the more stable average energy is obtained at 2.0 ps window because the fluctuations in average energy between positive and negative were less than 0.1 eV. Therefore, we took the average energy of the last 2.0 ps as the energy of CO_3_^•−^ in Supplementary Table 2, which also applies to the other three intermediates. According to the AIMD simulation results, here we suggest a carbonate-mediated 2e^−^ water oxidation reaction mechanism as shown in Fig. 4b (all these elementary reactions are exothermic under the electrochemical potential range we operated): First, CO_3_^2−^ is oxidized to CO_3_^•−^ at high oxidation potentials on the electrode surface (CO_3_^2−^ → CO_3_^•− ^+ e^−^, Δ*G* = − 0.72 eV at U = 3.0 V *vs*. RHE). Second, the generated CO_3_^•−^ coupled with a H_2_O molecule is further oxidized to HCO_4_^−^ (CO_3_^•− ^+ H_2_O → HCO_4_^− ^+ H^+^ + e^−^, Δ*G* = − 0.27 eV at U = 3.0 V *vs*. RHE). Subsequently, HCO_4_^−^ is hydrolyzed to generate H_2_O_2_ and converted back to HCO_3_^−^ or CO_2_ (HCO_4_^− ^+ H_2_O → HCO_3_^− ^+ H_2_O_2_ or HCO_4_^− ^+ H_2_O → CO_2_ + H_2_O_2_ + OH^−^, Δ*G* = − 0.31/0.42 eV without applied potential). As shown in Supplementary Fig. 12, the first and second elementary reactions are electrochemical steps. Under an electrode potential of 3.0 V, both reactions are exothermic. The third fundamental reaction is a non-electrochemical step, and the calculation results show that the former is more likely to occur than the latter. During this reaction step, H^+^ in H_2_O combines with the –OOH group in HCO_4_^−^ molecule to generate H_2_O_2_, and the remaining OH^−^ group from H_2_O can combine with remaining CO_2_ from HCO_4_^−^ to form HCO_3_^−^. We note here that this hydrolysis step involves O exchange between water and carbonate ions, which becomes an important evidence to be confirmed by experiments. Finally, HCO_3_^−^ or CO_2_ + OH^−^ generated from the anode, coupled with the remaining OH^−^ group generated from the cathode, is concerted back to CO_3_^2−^ to close the mediation loop in the system (Supplementary Fig. 13). We also found out that the FTO electrode provides a conducting and stable surface to extract electrons from carbonate, and may not serve as a classic catalytic surface where proper chemical bonds with reaction intermediates were usually established to facilitate reaction steps (Supplementary Figs. 14 and 15).

To support the proposed reaction mechanism, we first tested the formation of carbonate radical by electron paramagnetic resonance (EPR). Due to the short lifetime of carbonate radical, we used 5,5-dimethyl-1-pyrroline N-oxide (DMPO) as a spin trap and an in-situ trapping method (see Methods) to detect its generation under electrolysis (Supplementary Fig. 16a)^49^. Compared to the direct mixture of DMPO and before/post-electrolysis carbonate solution, the solution obtained from the in-situ trapping method under electrolysis exhibit a clear four-line 1:2:2:1 splitting pattern characteristic of the DMPO^•^ − OH adduct (Fig. 4c and Supplementary Fig. 16a), indicating that carbonate radical was formed at the surface of FTO electrode under oxidation potentials. Furthermore, we performed an ^18^O isotope labeling experiment to support the possible formation of HCO_4_^−^ intermediates. As we mentioned earlier, the hydrolysis process of HCO_4_^−^, if existing in the electrolyte, will lead to oxygen exchange between carbonate and water (Supplementary Fig. 16b). Therefore, if we use ^18^O isotope-labeled water as the electrolyte, we would expect an increased abundance of ^18^O (δ^18^O) in Na_2_CO_3_ after electrolysis compared to that of natural exchange without electrolysis (Methods)^11^. Two sets of ^18^O isotope experiments were therefore designed: First, to figure out the natural exchange rate as the background, we tested δ^18^O as a function of time in Na_2_CO_3_ after it was dissolved in ^18^O labeled water; Next, we did the same tests of δ^18^O at the same time points under WOR electrolysis. As clearly shown in Fig. 4d, the abundance of ^18^O isotope in Na_2_CO_3_ under electrolysis condition continues to increase over time, and is several orders of magnitude higher than that without electrolysis, suggesting the violent interaction and chemical bond reconfiguration between carbonate and water via the percarbonate intermediate pathway, instead of a direct water oxidation pathway (Supplementary Fig. 17).

In conclusion, we demonstrated a CO_2_/carbonate mediated electrochemical water oxidation for high-performance H_2_O_2_ generation, where carbonate ions help to steer the 4e^−^ reaction pathway to 2e^−^ via intermediates such as carbonate radicals and percarbonate, and FTO electrode provides a highly conductive, stable, and 4e^−^-OER inert surface. As a result, we achieved a high H_2_O_2_ selectivity of up to 87%, industrial-relevant H_2_O_2_ generation partial current of up to 1.3 A cm^−2^, as well as excellent stability, suggesting a promising route for the renewable and onsite generation of H_2_O_2_ using electricity. This mediation process could be further extended to other electrochemical oxidation applications, especially for those suffering from 4e^−^-OER competition such as chlorine evolution, hydrocarbon oxidation, nitrogen oxidation, etc. Future works can be focused on exploring other catalytic materials enabling this mediation reaction, improving the onset potentials, and further extending the durability.

## Methods

### Materials

FTO was purchased from MSE Supplies (2.2 mm 7-8 Ohm/Sq FTO TEC 7 Coated Glass Substrates, used in all experiments if there is no other noting) and Sigma (SKU: 735159 and 735256). AZO and ITO were purchased from Sigma. Titanium mesh was purchased from Kunshan GuangJiaYuan new materials Co. Ltd. The oxidized carbon was prepared according to a previous report^40^. Hydrophobic carbon fiber paper (CFP, PTFE loading 60*wt*.%) was purchased from Fuel Cell store. Chemicals were purchased from Sigma and VWR International Company.

### Electrocatalytic oxidation of H_2_O^11^

The electrochemical measurements were run at 25 °C in a customized gas-tight H-type glass cell separated by Nafion 117 membrane (Fuel Cell Store). A BioLogic VMP3 workstation was employed to record the electrochemical response. In a typical three-electrode system, a platinum foil (Beantown Chemical, 99.99%) and a saturated calomel electrode (SCE, CH Instruments) were used as the counter and reference electrode, respectively. The FTO electrodes were used as the working electrodes, and stainless-steel alligator clip was used to connect the FTO glass electrode. The alligator clip was not immerged in the electrolyte. The geometric area of the FTO electrode was 0.5 to 1 cm^2^, which was precisely defined by an electrochemically inert, hydrophobic wax (Apiezon wax WW100) during electrochemical tests. Before electrochemical measurements, all samples were pre-stabilized at 20 mA cm^−2^ to clean the FTO surface. All potentials measured against SCE (E_SCE_) were converted to the RHE (E_RHE_) scale in this work using E_RHE_ = E_SCE_ + 0.244 V + 0.0591 × pH, where pH values of the electrolytes were determined by an Orion 320 PerpHecT LogR Meter (Thermo Scientific). The pH values of investigated electrolytes are listed in Supplementary Table 2. The electrolyte in the anodic compartment was stirred at a rate of 1,600 r.p.m. during electrolysis. All the measured potentials were manually compensated unless stated otherwise. The overall resistance was determined by potentiostatic electrochemical impedance spectroscopy at frequencies ranging from 0.1 Hz to 200 kHz, and manually compensated as *E* (iR corrected versus RHE, where iR is the voltage drop from overall resistance) = *E* (versus RHE) – R (the overall resistance, including the electrolyte resistance and the intrinsic resistance of the electrode) × i (amps of average current). The impedance number was about 12 to 17 Ω. DC stabilized power supply (ITECH) was used for the ultra-high current density (over 1 A cm^−2^) electrolysis. The volume of the electrolyte solution was 25 mL, and the reaction time was determined by the electrolysis current to insured the total electrolysis coulomb was about 10 to 50 C. After electrolysis, the generated H_2_O_2_ was detected by using the standard potassium permanganate (0.1 N KMnO_4_ solution, Sigma-Aldrich) titration process, according to the following equation:

$$2{{{{{{\rm{MnO}}}}}}}_{4}^{-}+5{{{{{{\rm{H}}}}}}}_{2}{{{{{{\rm{O}}}}}}}_{2}+6{{{{{{\rm{H}}}}}}}^{+}\to 2{{{{{{\rm{Mn}}}}}}}^{2+}+5{{{{{{\rm{O}}}}}}}_{2}+8{{{{{{\rm{H}}}}}}}_{2}{{{{{\rm{O}}}}}}$$

The typical quantification time is about 5~10 min, which was much longer than the lifetime of carbonate radical (the lifetime of radical is about several microseconds)^50^.

In this work, sulfuric acid (2.0 N H_2_SO_4_, VWR International Company) was used as the H^+^ source. The FE for H_2_O_2_ production is calculated using the following equation:

$${{{{{\rm{FE}}}}}}=\frac{{{{{{\rm{generated}}}}}}\;{{{{{{\rm{H}}}}}}}_{2}{{{{{{\rm{O}}}}}}}_{2}\,\left({{{{{\rm{mol}}}}}}\right)\times 2\times 96485\,\left({{{{{\rm{C}}}}}}\;{{{{{{\rm{mol}}}}}}}^{-1}\right)}{{{{{{\rm{total}}}}}}\;{{{{{\rm{amount}}}}}}\;{{{{{\rm{of}}}}}}\;{{{{{\rm{charge}}}}}}\;{{{{{\rm{passed}}}}}}\;({{{{{\rm{C}}}}}})}\times 100\;({{{{{\rm{maximum}}}}}}\;100 \% )$$

For the stability test, a continuous three-electrodes flow cell was employed to continuously produce H_2_O_2_ (Supplementary Fig. 8b). The electrolyte in the anodic compartment was stirred at a rate of 1600 r.p.m. during electrolysis. A peristaltic pump (Longer) was used to pump in K_2_CO_3_ electrolyte to the WOR side, and another peristaltic pump was used to pump out generated H_2_O_2_ in the WOR side. The electrolyte flow rate of the WOR side was 6 mL min^−1^. The FE for H_2_O_2_ production is calculated using the following equation:

$${{{{{\rm{FE}}}}}}=\, 	\frac{{{{{{\rm{generated}}}}}}\;{{{{{{\rm{H}}}}}}}_{2}{{{{{{\rm{O}}}}}}}_{2}\,\left({{{{{\rm{mol}}}}}}{{{{{{\rm{L}}}}}}}^{-1}\right)\times 2\times 96485\;\left({{{{{\rm{C}}}}}}\;{{{{{{\rm{mol}}}}}}}^{-1}\right)\times {{{{{\rm{flow}}}}}}\;{{{{{\rm{rate}}}}}}\;({{{{{\rm{mL}}}}}}\,{{{{{{\rm{s}}}}}}}^{-1})}{{j}_{{{{{{\rm{total}}}}}}}({{{{{\rm{mA}}}}}})}\\ 	 \times 100\;({{{{{\rm{maximum}}}}}}\;100 \% )$$

For the 2e^−^-WOR//2e^−^-ORR electrosynthetic cell test, 0.5 mg cm^−2^ oxidized carbon catalyst was air-brushed onto 2-cm^2^ Sigracet 35 BC gas diffusion layer (Fuel Cell Store) electrodes as 2e^−^-ORR cathode. Then, a 1-cm^2^ FTO electrode was used as anode. The two electrodes were therefore placed on opposite sides in the H cell. O_2_-saturated 2.0 M Na_2_CO_3_ was used as electrolyte. The cathode was open to the atmosphere. The flow rate of 2.0 M Na_2_CO_3_ electrolyte was 3 ml min^−1^ at both sides, as controlled by a peristaltic pump. A current of 100 mA was employed for H_2_O_2_ production. The FE of the electrosynthetic cell for H_2_O_2_ production is calculated using the following equations, respectively:

$${{{{{\rm{FE}}}}}}=\frac{{{{{{\rm{generated}}}}}}\;{{{{{{\rm{H}}}}}}}_{2}{{{{{{\rm{O}}}}}}}_{2}\;\left({{{{{\rm{mol}}}}}}\;{{{{{{\rm{L}}}}}}}^{-1}\right)\times 2\times 96485\;\left({{{{{\rm{C}}}}}}\;{{{{{{\rm{mol}}}}}}}^{-1}\right)\times {{{{{\rm{flow}}}}}}\;{{{{{\rm{rate}}}}}}\;({{{{{\rm{mL}}}}}}\,{{{{{{\rm{s}}}}}}}^{-1})}{{j}_{{{{{{\rm{total}}}}}}}({{{{{\rm{mA}}}}}})}\times 100\;({{{{{\rm{maximum}}}}}}\;200 \% )$$

To obtain solid H_2_O_2_, the electrolyte after electrolysis (two-electrode configuration) was firstly concentrated by rotary evaporator, then about 100 ml of absolute isopropanol is added into 20 ml of the electrolyte for extraction, and the mixture is mechanically stirred. The precipitate is separated by vacuum filtration and washed several times with absolute isopropanol. The isolated precipitate is then dried in a vacuum oven at room temperature for 24 h.

To quantify the gas products during electrolysis, argon gas (Airgas, 99.995%) was delivered into the anodic compartment at a rate of 50.0 standard cubic centimeters per minute (sccm, monitored by an Alicat Scientific mass flow controller) and vented into a gas chromatograph (Shimadzu gas chromatography-2014) equipped with a combination of molecular sieve 5 Å, Hayesep Q, Hayesep T and Hayesep N columns. A thermal conductivity detector was mainly used to quantify gas product concentration. The partial current density for the O_2_ produced was calculated as follows:

$${j}_{i}={x}_{i}\times v\times \frac{{n}_{i}{{{{{\rm{F}}}}}}{p}_{o}}{{{{{{\rm{RT}}}}}}}\times {\left({electrode}\;{area}\right)}^{-1}$$

where *x*_*i*_ is the volume fraction of certain product determined by online GC referenced to calibration curves from the standard gas sample (Airgas), *v* is the flow rate of 50.0 sccm, *n*_*i*_ is the number of electrons involved, *p*_o_ = 101.3 kPa, F is the Faradaic constant, T= 298 K and R is the gas constant. The corresponding Faradaic efficiency at each potential is calculated by ${{{{{\rm{FE}}}}}}=\frac{{j}_{i}}{{j}_{{total}}}\times 100 \%$. Of note, H_2_O_2_ FEs and O_2_ FEs are evaluated from two completely different quantification systems: one is from solution titration, and another is from gas chromatographic method. When adding together, as shown in Fig. 2e, the total FEs for H_2_O_2_ and O_2_ are close to 100% (within testing error range), suggesting there are no side reactions other than WOR under the electrolysis conditions.

### Characterizations

SEM was performed on a FEI Quanta 400 field emission scanning electron microscope. Powder X-ray diffraction data were collected using a Bruker D2 Phaser diffractometer in parallel beam geometry employing Cu Kα radiation (λ = 1.54056 Å) and a 1-dimensional LYNXEYE detector, at a scan speed of 0.02° per step and a holding time of 1 s per step. X-ray photoelectron spectroscopy was obtained with a PHI Quantera spectrometer, using a monochromatic Al Kα radiation (1486.6 eV) and a low energy flood gun as neutralizer. All XPS spectra were calibrated by shifting the detected carbon C 1 s peak to 284.6 eV.

### ^18^O isotope measurement

We used ^18^O isotope-labeled water to prepare the Na_2_CO_3_ electrolyte. First, 0.14 mol (14.8 g) Na_2_CO_3_ (Sigma) was dissolved into 70 ml of ultrapure Milli-Q water. Then, 0.6 ml 10% H_2_O^18^ was added. After stirring for 5 min, the fresh 2.0 M Na_2_CO_3_ solution was used as the electrolyte for water oxidation. After electrolysis at 400 mA for a certain time, 1 ml of the electrolyzed 2.0 M Na_2_CO_3_ solution was taken out; meanwhile another 1 ml of solution was taken from the bulk 2.0 M Na_2_CO_3_ solution without electrolysis. Then both of the sample solutions were quickly precipitated by 5 ml 1.0 M BaCl_2_. Next, the solution was centrifuged and washed with ultrapure Milli-Q water. The powder was then dried at 60 °C for ^18^O isotope analysis. Of note, the natural exchange time with the water was the same for the unelectrolyzed and electrolyzed Na_2_CO_3_. Oxygen isotope analysis was performed on a Gas Bench-Conflo-Isotope Ratio Mass Spectrometer (Delta V, Thermo Scientific) system. Then ~0.2 mg of sample powder was weighed out into an exetainer, which was flushed by helium flow for 10 min on the GasBench. Then 0.3 ml of 105% phosphoric acid was added into the exetainer for 24-h reaction. The generated CO_2_ gas was then delivered to the mass spectrometry for isotope ratio analysis. The oxygen isotope composition of the carbonate was calculated based on the measured oxygen isotope composition of the CO_2_ gas, based on the fractionation factor between the two at the reaction temperature. The isotope experiments to monitor the ^18^O isotope offers an analytical precision (1σ) of 0.05‰ for δ^18^O and the values are reported as standard δ notation with respect to the Vienna Standard Mean Ocean Water (VSMOW)^51^.

### EPR measurement

Electrochemical generated carbonate radical was performed in 1.0 M Na_2_CO_3_ solution with a constant electrolysis current of 200 mA. Once the potential became stable, pipette contains DMPO solution (200 mM, 100 μl) was immersed into the electrolyte and make sure the pipette tip was fully contact with the FTO electrode. The radical generated on the electrode will in-situ react with the DMPO solution in the interface between pipette and FTO electrode, and the final solution (sample named solution obtained from in-situ trapping method) contains 100 μl electrolyte and 100 μl DMPO solution. For the sample named DMPO + Na_2_CO_3_ solution, DMPO solution (200 mM, 100 μl) was directly mixed with 1.0 M Na_2_CO_3_ (100 μl). For the sample named DMPO + post-electrolysis solution, DMPO solution (200 mM, 100 μl) was mixed with the electrolyte (100 μl) after electrolysis at 200 mA for 5 min. Finally, above obtained solutions were transferred to capillary for EPR test. All EPR measurements were taken at room temperature with EPR spectrometer (Bruker A300). We set typical parameters as follows: 3515 G center of field; 9.852054 GHz frequency; 1 G modulation amplitude; 40 msec conversion time; 81.92 msec time constant; 20.38 Mw microwave power; 120 G scan width; 40 s sweep time.

### Molecular dynamic calculations

A cubic box 12 Å × 12 Å × 12 Å was modeled with 57 water molecules to maintain a density of water at ∼1 g cm^−3^ (Supplementary Fig. 11b). To fit the box of this size, CO_3_^•−^, HCO_4_^−^, HCO_3_^−^ + H_2_O_2_ and CO_2_ + H_2_O_2_ + OH^−^ replaced seven, seven, eight, and eight water molecules, respectively. The negative charge (^−^) of the ion was neutralized by a uniform background charge. We did not consider the hydroxide ions and protons in the electrolyte during simulation. Total energy of the above four possible intermediates was calculated by AIMD at a constant temperature of 300 K (using Nose-Hoover thermostat^52^, with a time step of 0.5 fs) as implemented in the Vienna ab initio simulation program (VASP)^53,54^. Perdew-Burke-Ernzerhof (PBE) functional^55^ and DFT-D3^56^ methods were used to describe the exchange and correlation energies and the van der Waals interactions, respectively. A plane-wave cutoff energy of 400 eV and Gamma centered k-mesh of 1 × 1 × 1 were set in MD simulations. Considering the calculation cost, we ran 7.5 ps for each structure. From these AIMD calculations, we analyzed the arithmetic average of their total energies using different averaging time windows from 0.5 ps to 2.0 ps. It is found that averaging within a time window of 2.0 ps gives a value fluctuates around the final value by ±0.1 eV, less than other time windows. Therefore, average energy was evaluated from last 2.0 ps AIMD trajectory, as shown in Supplementary Table 3. The formation of CO_3_^•−^ and HCO_4_^−^ are electrochemical oxidation steps. Supplementary Table 4 shows inorganic standard electrode potentials^45^. The reaction between H_2_O and CO_3_^•−^ was calculated by two separated process including H_2_O → OH^•^ + H^+^ + e^−^ and CO_3_^•−^ + OH^•^ → HCO_4_^−^ because the energy of H^+^ + e^−^ item in CO_3_^•−^ + H_2_O → HCO_4_^−^ + H^+^ + e^−^ reaction step is difficult to accurately calculate by MD simulations. Therefore, we deal with this step by adding the above two steps together. To better match the results of the experiment, we convert the standard electrode potential into a reversible hydrogen electrode (U_RHE_ = U_SHE_ + 0.059 × pH, pH = 12). Take CO_3_^2−^ → CO_3_^•−^ + e^−^ reaction as an example, the standard electrode potential for this reaction is +1.57 ± 0.03 V and the potential corresponding to conversion to RHE is 2.278 V (pH=12, U_RHE_ = + 1.57 + 0.059 × 12 = 2.278 V), corresponding to the reaction free energy of 0 eV. When the electrode potential is increased to 3 V, the reaction is exothermic with an energy of −0.72 eV.

### Gibbs free energies on the SnO_2_(110) surface calculations

The exchange-correlation potential is described by the generalized gradient approximation (GGA) with spin polarized revised Perdew-Burke-Ernzerhof (RPBE)^57^ functional due to that it is more adept at describing chemisorption on metals. The projector augmented wave is applied to describe the electron-ion interaction and the plane-wave energy cutoff is set to 400 eV. All structures are optimized with a convergence criterion of 1 × 10^−5^ eV for the energy and 0.02 eV/Å for the forces. The SnO_2_(110) surface is the most common of the faces of rutile structure tin oxide (Space group: P42/mnm, No. 136)^58^. Therefore, the SnO_2_(110) surface is modeled using a slab with a (4 × 2) surface supercell consisting of 3 trilayers and containing 144 atoms. The vacuum spacing is set to more than 15 Å for surface isolation to prevent interaction between two neighboring surfaces. The top two trilayers are fully relaxed during the structural optimization and geometry optimizations for SnO_2_(110) are performed with 1 × 1 × 1 k-mesh. The Poisson-Boltzmann (PB) implicit solvation model, Vaspsol^59^, was used to describe the effect of solvation as implemented in VASP 5.4.4, with a dielectric constant *ε* = 80 for water. All molecules were performed in a 15 × 15 × 15 Å^3^ unit cell with a 5 × 5 × 5 k-point grid sampling.

Computational hydrogen electrode (CHE)^60^ model was used to calculate the Gibbs free energy change for OER elementary reactions. At electrode potential *U* = 0 V (vs. reversible hydrogen electrode, RHE), the Gibbs free energy change (Δ*G*) can be calculated by

$$\Delta {{G}}=\Delta {{E}}+\Delta {{{E}}}_{{{ZPE}}}-{{T}}\Delta {{S}}+\Delta {{{G}}}_{{{U}}}$$

where Δ*E* is the energy difference between the products and reactants from DFT computations; Δ*E*_ZPE_ and Δ*S* are the changes in zero-point energy and entropy, respectively, which are obtained from the vibrational frequency calculations; *T* is the temperature at 298 K. The energy corrections of gas-phase species in this work, including zero point energies (ZPE) and entropies (TS), are listed in Supplementary Table 5. Δ*G*_U_ = −*neU*, where U is the electrode applied potential relative to RHE, *e* is the elementary charge transferred and *n* is the number of proton–electron pairs transferred. 2e^–^-WOR path is calculated in alkaline condition. We calculated the chemical potential of hydroxides and electrons with reference to previous work^61^. μ(e^–^)– μ(OH^–^) – *eU* = 9.81 eV was obtained by repeated calculations under reversible hydrogen electrode potential (*U*_0_(RHE) =1.23 V) at *T* = 298.15 K.

The accuracy of the above DFT methodology applied in our work is mainly based on published work. More details can be found in the Supporting Information.

## Supplementary information

Supplementary Information

Peer Review File

## Data Availability

The data that support the findings of this study are available from the corresponding authors upon reasonable request.
