# Supplementary material for: CO2/carbonate-mediated electrochemical water oxidation to hydrogen peroxide
Source: Nat Commun. 2022 May 13;13:2668. doi: 10.1038/s41467-022-30251-5 (PMC9106728; doi:10.1038/s41467-022-30251-5)
Supplement: Supplementary file 1 — Supplementary Information [file 41467_2022_30251_MOESM1_ESM.pdf]

Supplementary Information for

CO_2/carbonate-mediated electrochemical water oxidation to hydrogen peroxide

Lei Fan[1,2]†, Xiaowan Bai[3]†, Chuan Xia[1,4]†, Xiao Zhang[1], Xunhua Zhao[3], Yang Xia[1], Zhen-Yu Wu[1], Yingying Lu[2]*, Yuanyue Liu[3]*, Haotian Wang[1,5,6]*

[1]Department of Chemical and Biomolecular Engineering, Rice University, Houston, TX 77005, USA.
[2]State Key Laboratory of Chemical Engineering, Institute of Pharmaceutical Engineering, College of Chemical and Biological Engineering, Zhejiang University, Hangzhou 310027, China.
[3]Texas Materials Institute and Department of Mechanical Engineering, The University of Texas at Austin, Austin, Texas 78712, USA.
[4]Smalley-Curl Institute, Rice University, Houston, TX 77005, USA.
[5]Department of Materials Science and NanoEngineering, Rice University, Houston, TX 77005, USA.
[6]Department of Chemistry, Rice University, Houston, TX 77005, USA.
†These authors contributed equally.
*Corresponding author Emails: yingyinglu@zju.edu.cn (Y.L.); Yuanyue.liu@austin.utexas.edu (Y.L.); htwang@rice.edu (H.W.).

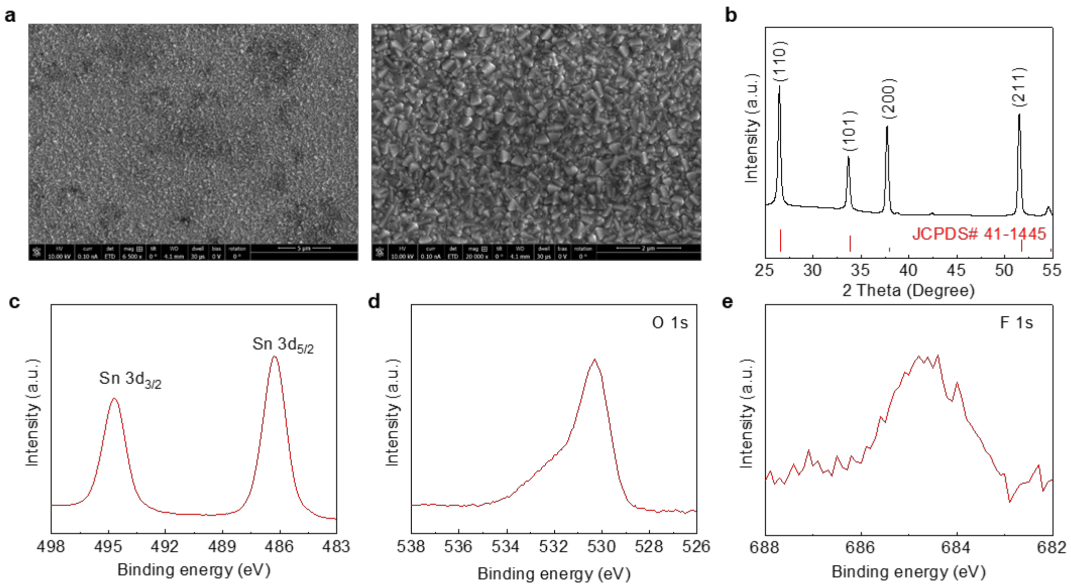

Supplementary Fig. 1 | Characterizations of FTO electrode. a, SEM images of FTO electrode. **b,** XRD pattern of FTO electrode. **c-e,** XPS spectral of FTO electrode.

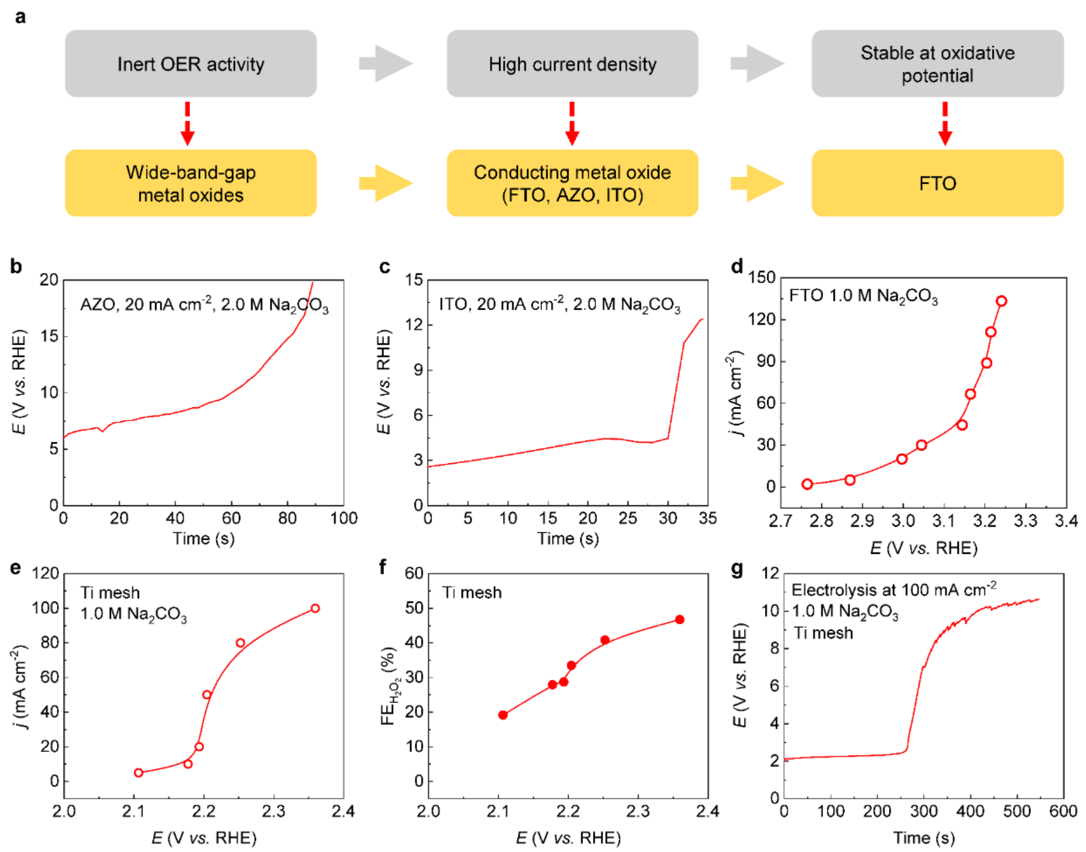

Supplementary Fig. 2 | Screening of different catalytic electrodes. a, Catalytic electrode selection process. First, to achieve high-selectivity H_2O_2 generation, we need to use OER-inert catalyst. While most transition metal oxide catalysts show high OER activity, wide-band-gap metal oxides are usually inert for OER. However, most wide-ban-gap metal oxides exhibit low electron conductivity, which is difficult to achieve a high current density. To achieve high productivity (current density), we can choose conducting metal oxides, such as fluorine-doped tin oxide (FTO), aluminum-doped zinc (AZO) oxide, and indium tin oxide (ITO). Finally, as shown in (**b**) and (**c**), AZO and ITO are unstable under oxidative potentials. FTO showed inert OER activity (**d**), high electron conductivity, and high stability under WOR potentials (**Fig. 3e**), which makes it a promising electrode candidate for this carbonate-mediated $2e^-$-WOR. **b,** Electrochemical potential of AZO as a function of time by maintaining a 20 mA cm^{-2} WOR current. **c,** Electrochemical potential of ITO as a function of time by maintaining a 20 mA cm^{-2} WOR current. **d,** I-V curves of FTO in 1.0 M Na_2CO_3, the onset potential for water oxidation was about 2.75 V *vs.* RHE, which was much higher than 1.23 V, indicating the inert OER performance of FTO. **e-f,** I-V curves of titanium mesh in 1.0 M Na_2CO_3 and corresponding H_2O_2 FEs. **g,** Electrochemical potential of titanium mesh as a function of time by maintaining a 100 mA cm^{-2} WOR current in 1.0 M Na_2CO_3. Due to the inert four electron water oxidation reaction, titanium mesh also exhibited high selectivity towards H_2O_2 with the promotion effects of carbonate. However, the metallic titanium surface will be gradually passivated under WOR potentials, leading to the degradation of its catalytic performance and limiting its for practical application.

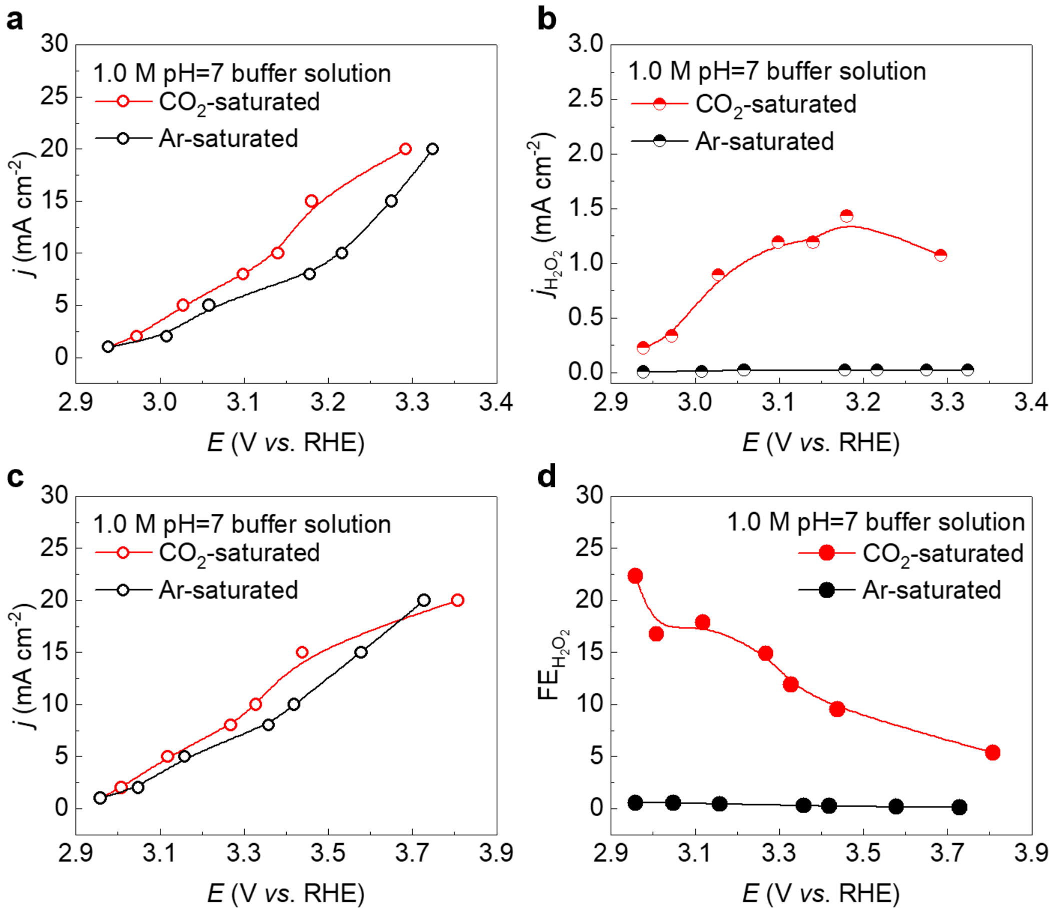

Supplementary Fig. 3 | Impacts of CO_2 on electrochemical H_2O oxidation. a, I-V curves of FTO electrode using CO_2-saturated and Ar-saturated 1 M sodium phosphate buffer solution (pH ~ 7). **b,** Corresponding H_2O_2 partial current densities at different potentials. **c-d**, I-V curves and corresponding H_2O_2 FEs of FTO electrode using CO_2-saturated and Ar-saturated 1 M sodium phosphate buffer solution (pH ~ 7) without iR compensation.

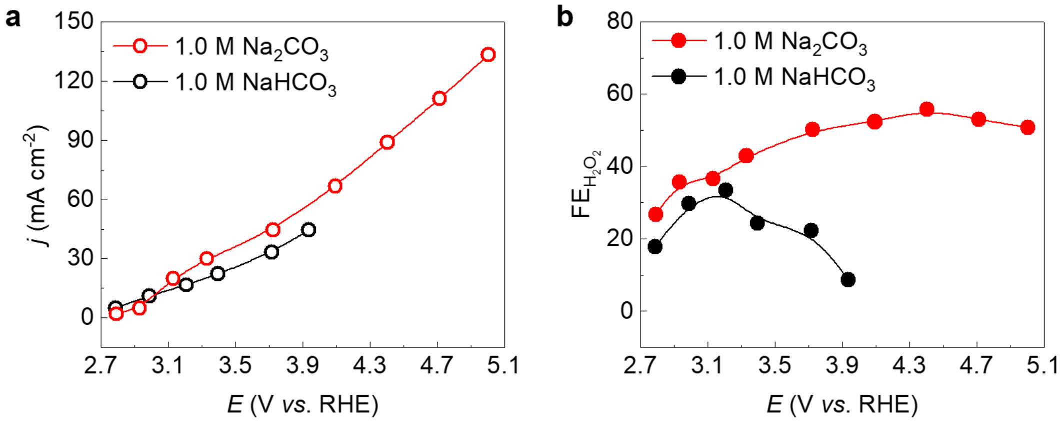

Supplementary Fig. 4 | I-V curves and corresponding H_2O_2 FEs in 1.0 M $NaHCO_3$ and 1.0 M Na_2CO_3 without iR compensation.

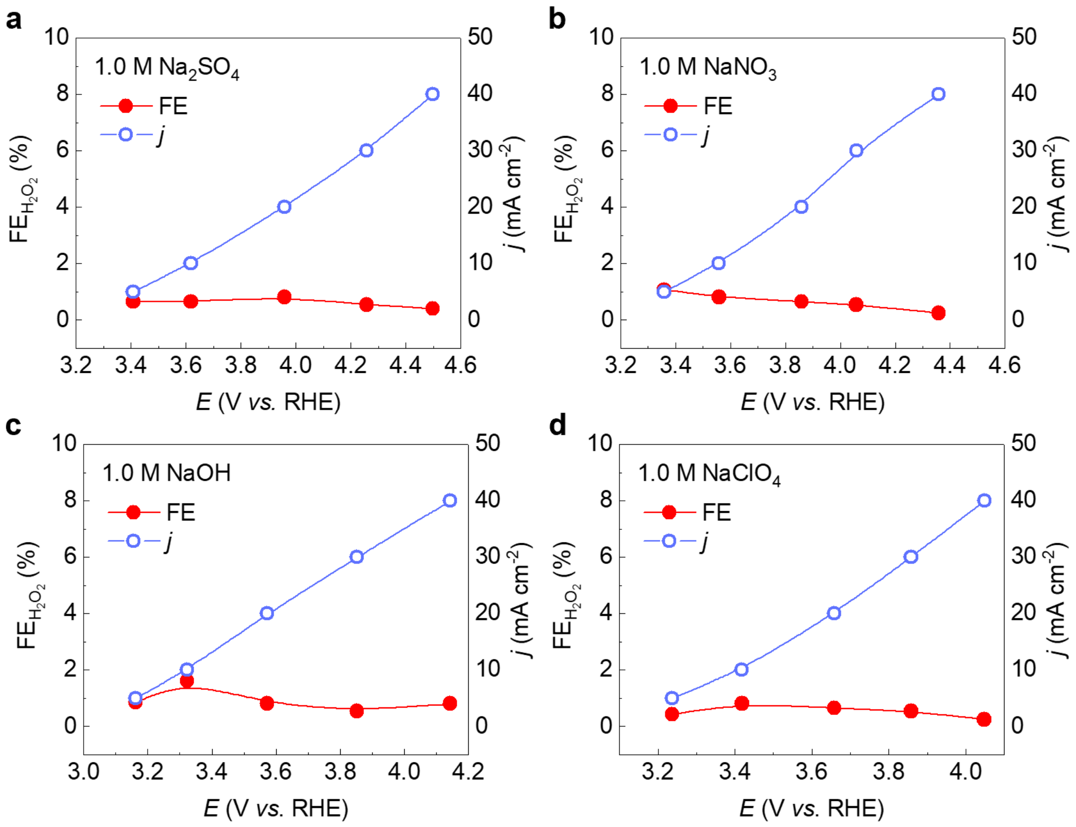

Supplementary Fig. 5 | I-V curves and H_2O_2 FEs of FTO catalyst using 1.0 M Na_2SO_4 (a), 1.0 M $NaNO_3$ (b), 1.0 M NaOH (c), and 1.0 M $NaClO_4$ (d) without iR compensation.

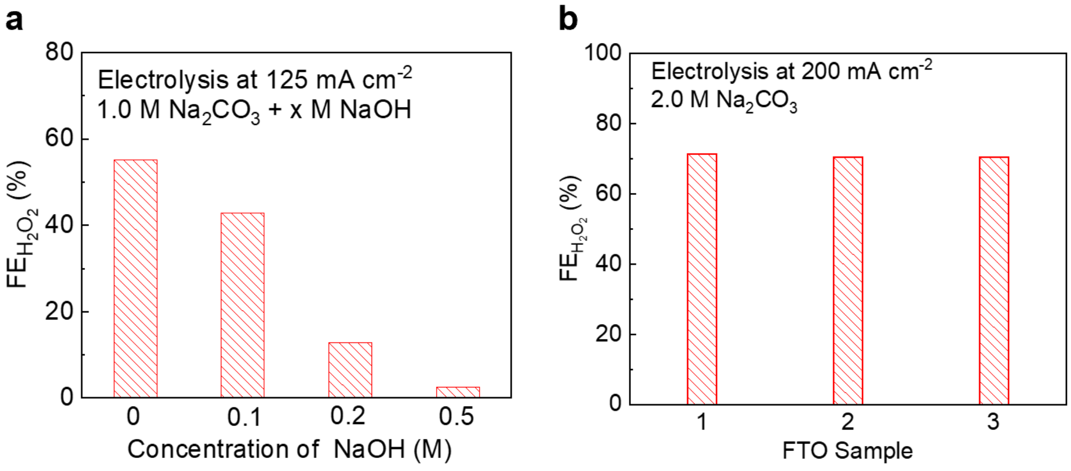

Supplementary Fig. 6 | Impacts of OH^- and FTO samples on electrochemical H_2O oxidation. a, H_2O_2 FE in 1.0 M Na_2CO_3 with different concentrations of NaOH. **b,** H_2O_2 FEs in 2.0 M Na_2CO_3 under 200 mA cm^{-2} using different types of FTO samples. Sample 1 was from MSE Supplies (2.2 mm 7-8 Ohm/Sq FTO TEC 7 Coated Glass Substrates); sample 2 was from and Sigma (SKU: 735159, surface resistivity ~7 Ω/sq); sample 3 was from Sigma (SKU: 735256, surface resistivity ~13 Ω/sq). Their similar H_2O_2 selectivity suggests that the carbonate mediation process is not sensitive to the varied properties of FTO electrode.

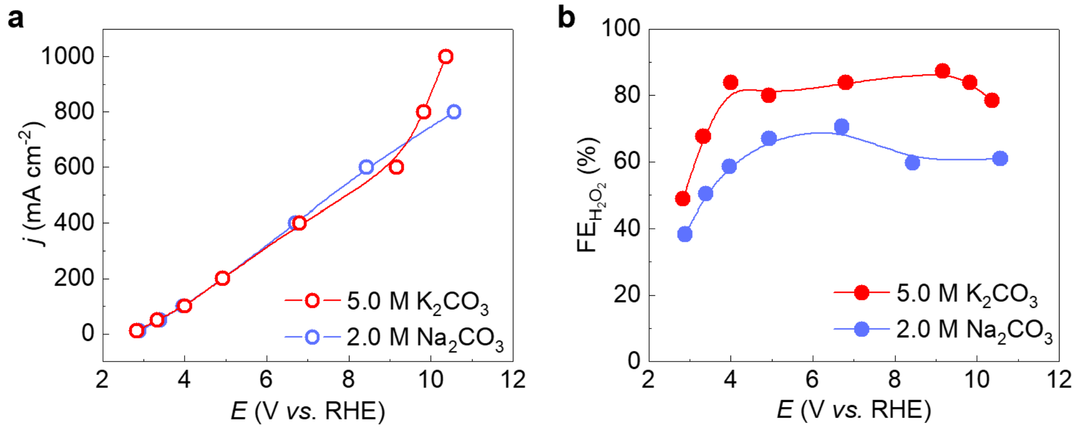

Supplementary Fig. 7 | I-V curves and corresponding H_2O_2 FEs in 2.0 M Na_2CO_3 and 5.0 M K_2CO_3 without iR compensation.

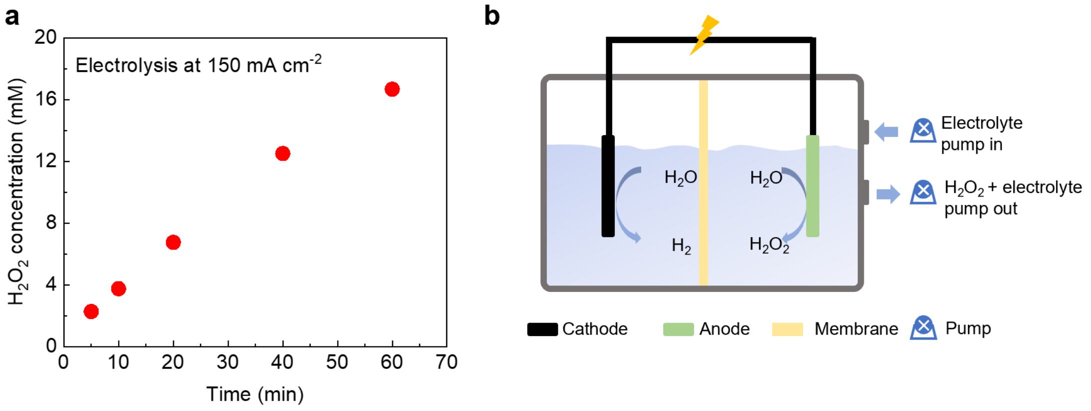

Supplementary Fig. 8 | Stability test of 2e$^-$-WOR. a, Time dependance of H_2O_2 concentration in traditional batch cell. **b,** Schematic illustration of a continuous flow reactor for long-term stability test. Our continuous flow reactor resolves the H_2O_2 accumulation challenge by maintaining a stable electrocatalysis environment for long term operation.

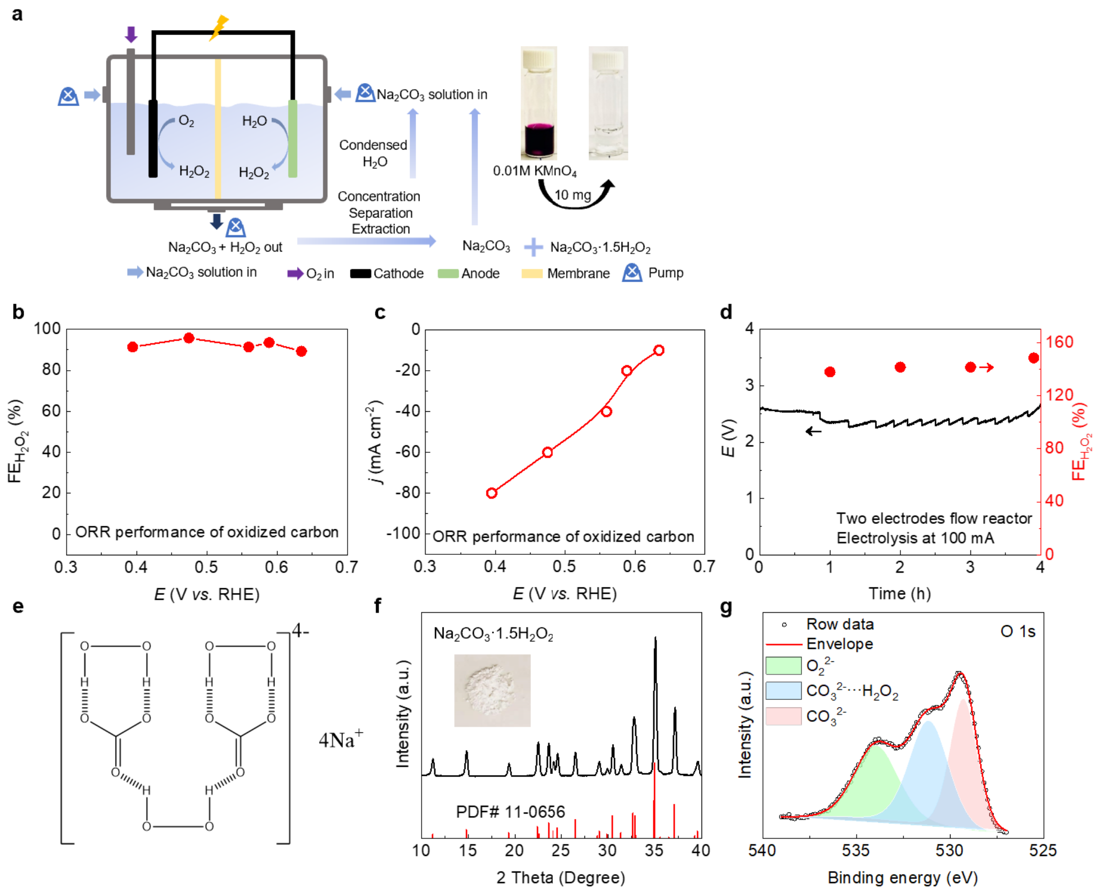

Supplementary Fig. 9 | Practical application of 2e$^-$-WOR. a, Schematic illustration of our H_2O_2 generation from both electrode by coupling 2e$^-$-WOR and 2e$^-$-ORR, together with the preparation of an adduct product between Na_2CO_3 and H_2O_2 ($Na_2CO_3{\cdot}1.5H_2O_2$). After electrolysis, the electrolyte solution was concentrated and separated to increase the H_2O_2 concentration in the Na_2CO_3 solution. The high concentration H_2O_2-Na_2CO_3 solution was then directly extracted to get pure solid $Na_2CO_3{\cdot}1.5H_2O_2$ powder. The right picture showed the degradation of $KMnO_4$ using as-obtained solid $Na_2CO_3{\cdot}1.5H_2O_2$ powder. **b,c,** I-V curve and corresponding H_2O_2 FEs of 2e$^-$-ORR using oxidized carbon catalyst in 2.0 M Na_2CO_3. **d,** Cell voltage and H_2O_2 FE of our 2e$^-$-ORR//2e$^-$-WOR cell as a function of time by fixing a cell current at 100 mA. **e,** Chemical structure of $Na_2CO_3{\cdot}1.5H_2O_2$. **f,** The XRD pattern for as-extracted $Na_2CO_3{\cdot}1.5H_2O_2$ from electrolyte after electrolysis. Inset is the photo of as obtained $Na_2CO_3{\cdot}1.5H_2O_2$. **g,** O 1s XPS spectrum of $Na_2CO_3{\cdot}1.5H_2O_2$. The solid-state $Na_2CO_3{\cdot}1.5H_2O_2$ can avoid the storage and transportation challenges of liquid-phase H_2O_2 solution because liquid-phase H_2O_2 solutions will happen self-accelerating decomposition reactions if there are any contaminants. In addition, liquid-phase H_2O_2 solutions exhibit high leakage risk during storage and transportation.

a

step #1: $H_2O \rightarrow OH^{\bullet} + H^+ + e^-$

step #2: $H_2O \rightarrow OH^{\bullet} + H^+ + e^-$

step #3: $OH^{\bullet} + OH^{\bullet} \rightarrow H_2O_2$

b

step #1: $H_2O \rightarrow OH^{\bullet} + H^+ + e^-$

step #2: $OH^{\bullet} + CO_3^{2-} \rightarrow CO_3^{\bullet-} + OH^-$

step #3: $CO_3^{\bullet-} + OH^{\bullet} \rightarrow HCO_4^-$

step #4: $HCO_4^- + H_2O \rightarrow HCO_3^- + H_2O_2$

step #5: $HCO_3^- \rightarrow H^+ + CO_3^{2-}$

c

step #1: $H_2O \rightarrow OH^{\bullet} + H^+ + e^-$

step #2: $OH^{\bullet} + CO_3^{2-} \rightarrow CO_3^{\bullet-} + OH^-$

step #3: $CO_3^{\bullet-} + CO_3^{\bullet-} \rightarrow C_2O_6^{2-}$

step #4: $C_2O_6^{2-} \rightarrow CO_4^{2-} + CO_2$

step #5: $CO_4^{2-} + H_2O \rightarrow CO_3^{2-} + H_2O_2$

d

step #1: $CO_3^{2-} \rightarrow CO_3^{\bullet-} + e^-$

step #2: $H_2O \rightarrow OH^{\bullet} + H^+ + e^-$

step #3: $CO_3^{\bullet-} + OH^{\bullet} \rightarrow HCO_4^-$

step #4: $HCO_4^- + H_2O \rightarrow HCO_3^- + H_2O_2$

step #5: $HCO_3^- \rightarrow H^+ + CO_3^{2-}$

e

step #1: $CO_3^{2-} \rightarrow CO_3^{\bullet-} + e^-$

step #2: $CO_3^{2-} \rightarrow CO_3^{\bullet-} + e^-$

step #3: $CO_3^{\bullet-} + CO_3^{\bullet-} \rightarrow C_2O_6^{2-}$

step #4: $C_2O_6^{2-} \rightarrow CO_4^{2-} + CO_2$

step #5: $CO_4^{2-} + H_2O \rightarrow CO_3^{2-} + H_2O_2$

f

step #1: $CO_3^{2-} \rightarrow CO_3^{\bullet-} + e^-$

step #2: $CO_3^{\bullet-} + OH^- \rightarrow HCO_4^- + e^-$

step #3: $HCO_4^- + H_2O \rightarrow HCO_3^- + H_2O_2$

step #4: $HCO_3^- \rightarrow H^+ + CO_3^{2-}$

g

step #1: $CO_3^{2-} \rightarrow CO_3^{\bullet-} + e^-$

step #2: $CO_3^{\bullet-} + H_2O \rightarrow HCO_4^- + H^+ + e^-$

step #3: $HCO_4^- + H_2O \rightarrow HCO_3^- + H_2O_2$

step #4: $HCO_3^- \rightarrow H^+ + CO_3^{2-}$

h

step #1: $CO_3^{2-} \rightarrow CO_3^{\bullet-} + e^-$

step #2: $CO_3^{\bullet-} + H_2O \rightarrow HCO_4^- + H^+ + e^-$

step #3: $HCO_4^- + H_2O \rightarrow CO_2 + H_2O_2 + OH^-$

step #4: $CO_2 + OH^- \rightarrow HCO_3^-$

$HCO_3^- \rightarrow H^+ + CO_3^{2-}$

Supplementary Fig. 10 | Screening of possible reaction pathways. Based on possible species existing in the electrolyte under the oxidation potentials we tested, we proposed several possible reaction pathways as listed in (**a-g**). **a,** H_2O is first oxidized to $OH^{\bullet}$, and then two $OH^{\bullet}$ coupled to form H_2O_2. If H_2O_2 generated from this pathway, then the anions would not have a direct impact on the H_2O_2 selectivity. However, as shown in our experimental results, we found out that carbonate ions would significantly change the WOR pathway. Additionally, the standard electrochemical potential of H_2O-to-$OH^{\bullet}$ conversion is +3.438 ± 0.017 V *vs.* RHE, which is much higher than the onset potentials (~ 2.75 V *vs.* RHE for FTO electrode and 2.1 V *vs.* RHE for titanium mesh) we detected H_2O_2 generation. As a result, we excluded this pathway, as well as pathways listed in **b-d,** involving the generation of $OH^{\bullet}$. **e,** In this proposed pathway, CO_3^{2-} is first oxidized to $CO_3^{\bullet-}$, and then two $CO_3^{\bullet-}$ coupled together to form $C_2O_6^{2-}$. The standard electrochemical potential of $CO_3^{\bullet-}$ formation from CO_3^{2-} oxidation is +2.278 ± 0.03 V *vs.* RHE, it's close to the onset potentials for H_2O_2 generation on FTO and titanium mesh. However, the following $CO_3^{\bullet-}$ coupling step is difficult due to the strong repulsion between two negatively charged ions. Therefore, this step is excluded. **f,** This mechanism also excluded due to the strong repulsion between two negatively charged ions ($CO_3^{\bullet-}$ and OH^-) during their coupling. Finally, we focused on pathways in (**g**) and (**h**) for further calculations.

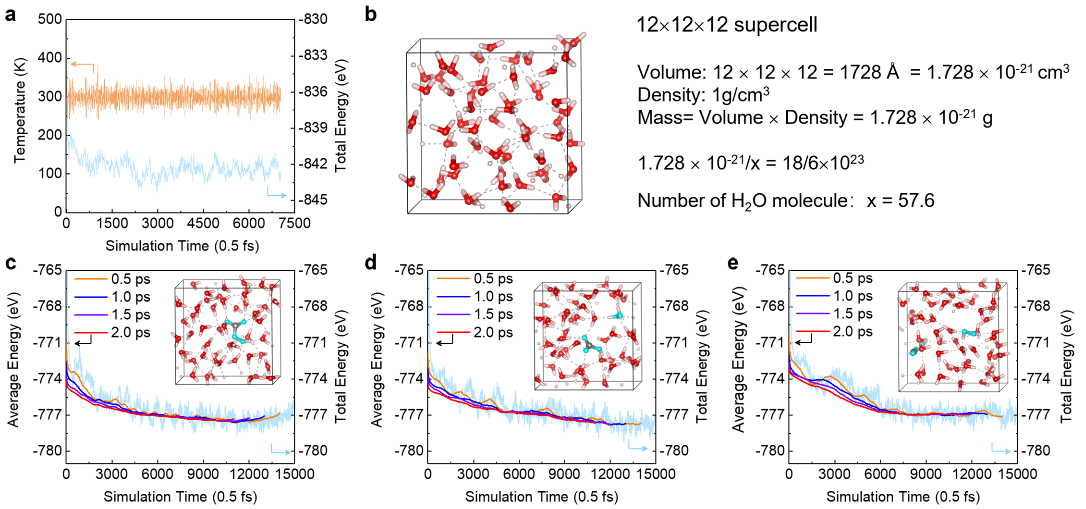

Supplementary Fig. 11 | Configuration of AIMD simulations and the convergence of average energy of key intermediates. For all the simulations, we use a cubic cell with 57 water molecules at density 1 g cm^{-3} (L = 12 Å). To maintain the density in the aqueous phase close to 1 g/cm^3, we removed seven or eight water molecules when adding four reaction intermediates, such as $CO_3^{\bullet-}$, HCO_4^-, HCO_3^- + H_2O_2, and CO_2 + H_2O_2 + OH^-. At this time, the corresponding carbonate concentration is 2%, which is consistent with the experimental carbonate concentration (~1.0 M Na_2CO_3). To demonstrate that the simulation results are robust with respect to the size of the simulation cell, we calculated the arithmetic average of their total energies using different averaging time window from 0.5 ps to 2.0 ps. It is found that averaging within a time window of 2.0 ps gives a value fluctuates around the final value by ±0.1 eV. Therefore, the averaging total energy obtained in a 2.0 ps simulation (after allowing the simulation to equilibrate, i.e., locate the local minima) was deemed sufficient. **a,** Variations of temperature and energy against the time for AIMD simulations of 57 H_2O molecules in a 12Å×12Å×12Å supercell. **b,** A snapshot of atomic configuration in the simulation. The simulation ran under 300 K for 3.5 ps with a time step of 0.5 fs. **c,** Convergence of average energy of HCO_4^- with 50 H_2O molecules. **d,** Convergence of average energy of HCO_3^- + H_2O_2 with 49 H_2O molecules. **e,** Convergence of average energy of CO_2 + H_2O_2 + OH^- with 49 H_2O molecules.

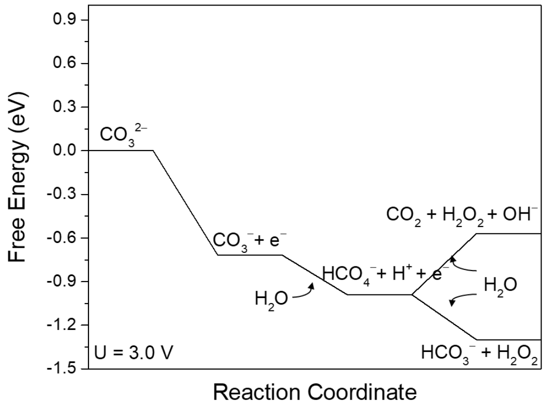

Supplementary Fig. 12 | The complete thermodynamic profile corresponding to reaction mechanism of carbonate-mediated 2e$^-$-WOR to H_2O_2. According to BEP principle, the activation energy is correlated with the reaction transition state energy decreases as the final state energy decreases[1] in our most favorable reaction mechanism, all elementary reaction steps are exothermic in thermodynamics, so we believe that the barriers of their transition states should be small at a potential up to ~3 V. Therefore, the activation energies were not calculated. Such calculations would be very valuable for making more definitive assessment on the reaction mechanism (for example, see our previous works on ORR[2] and CO_2RR[3], although the computational cost is extremely high for simulating the reaction kinetics of electrochemical interface.

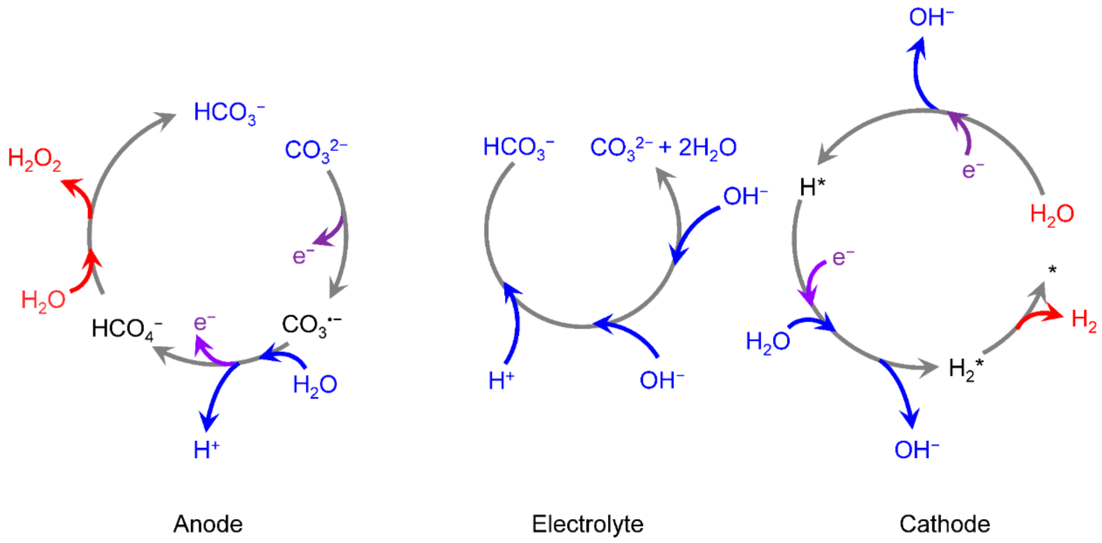

Anode reactions:
step #1: $CO_3^{2-} \rightarrow CO_3^{\bullet-} + e^-$
step #2: $CO_3^{\bullet-} + H_2O \rightarrow HCO_4^- + H^+ + e^-$
step #3: $HCO_4^- + H_2O \rightarrow HCO_3^- + H_2O_2$

Cathode reactions:
step #1: $H_2O + e^- \rightarrow H^* + OH^-$
step #2: $H^* + H_2O + e^- \rightarrow H_2^* + OH^-$
step #3: $H_2^* \rightarrow H_2 + *$

$$HCO_3^- + H^+ + OH^- + OH^- \rightarrow CO_3^{2-} + 2H_2O$$

Anode product: H_2O_2 Cathode product: H_2

Overall reaction: $2H_2O \rightarrow H_2O_2 + H_2$

Supplementary Fig. 13 | Elementary reaction steps on the anode and cathode.

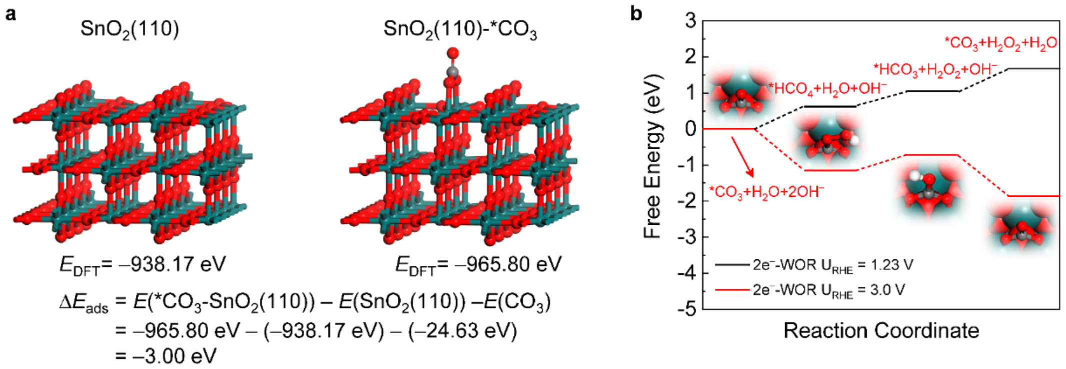

Supplementary Fig. 14 | Calculations of carbonate adsorption-based mechanism on FTO electrode. Based on our experimental studies, we concluded that carbonate must participate in the water oxidation process. Whether the FTO electrode could serve as a catalytic surface is unknown yet. Here we evaluated the possibility if the carbonate-mediated WOR could proceed via classic surface catalysis, which is that the carbonate and its following reaction intermediates are adsorbed on FTO surface via chemical bonds. Since the (110) surface is the most thermodynamically stable surface and has received extensive attention in previous experimental and theoretical studies, we chose the (110) surface of SnO_2 to study the surface reaction (For SnO_2(110) surface, half of the Sn atoms are bare and another half of the Sn atoms are O-terminated)[4–6]. If the surface is completely covered by CO_3, the distance between adjacent CO_3 adsorbates is > 4 Å, thus the impact on performance is relatively small. Here, we only consider one CO_3 adsorption on the Sn(110) surface for evaluating the oxygen exchange mechanism. **a,** The structures of SnO_2(110) and CO_3 adsorption on SnO_2(110) surface. The CO_3 is strongly adsorbed on SnO_2(110) surface with an adsorption energy of −3.00 eV based on DFT calculation by $\Delta E_{ads} = E(*CO_3\text{-}SnO_2(110)) - E(SnO_2(110)) - E(CO_3) = -965.80$ eV – (–938.17 eV) – (–24.63 eV) = – 3.00 eV. **b,** Possible surface reaction mechanism through carbonate adsorption. *Step #1*: OH^- is preferentially adsorbed on O atom in $*CO_3$ to form $*HCO_4$; *Step #2*: $*HCO_4$ further oxidizes H_2O to H_2O_2 forming $*HCOO_2$ (as $*HCO_3$). Here an O exchange occurs between HCO_4 and water; *Step #3*: $*HCOO_2$ combines with the second OH^- to generate a H_2O molecular and $*COO_2$ ($*CO_3$). The proposed reaction pathway is unfavorable for H_2O_2 formation under high oxidation potentials (U = 3.0 V *vs*. RHE) because the second step is endothermic by 0.43 eV, which is a non-electron transfer step, and thus the Gibbs free energy does not change with the electrode applied potential. In addition, CO_3 participated in the electrocatalytic process is strongly adsorbed on SnO_2(110) surface with an adsorption energy of −3.00 eV, which is difficult to desorb away from the surface, suggesting the possible surface passivation. This proposed surface catalysis reaction mechanism, if exists, is not the dominant reaction mechanism as it contradicts with our experimental observations. As the CO_3 is strongly adsorbed on FTO surface, the O exchange between water and carbonate would only take place at the beginning of the reaction, and therefore the abundance of ^{18}O in carbonate will not continuously increase over time, which is inconsistent with our experimental results (Fig. 4d). Considering the constant-potential method also suggest that the reaction is unlikely to occur on the surface, due to the

unfavorable thermodynamics for the non-electrochemical (i.e. thermal) step: $^{*}CO_2$-OOH + H_2O → $^{*}HCO_3$ + H_2O_2 in Supplementary Fig. 15. As a result, we concluded that in our system, H_2O_2 generation reaction may mainly occur in the electrolyte rather than on FTO surface, and FTO only serves as a stable, conducting and inert electrode for the extraction of electrons. The high H_2O_2 FE on titanium mesh also supports this conclusion as a side evidence.

Elementary Reaction	N_e	ΔG(eV)
(1) $^*CO_3 + OH^- \rightarrow {}^*CO_2\text{-}OOH$	1.38	-3.57
(2) $^*CO_2\text{-}OOH + H_2O \rightarrow {}^*HCO_3 + H_2O_2$	0.07	0.33
(3) $^*HCO_3 + OH^- \rightarrow {}^*CO_3 + H_2O$	0.55	-2.82

overall reaction: $2OH^- \rightarrow H_2O_2 + 2e^-$

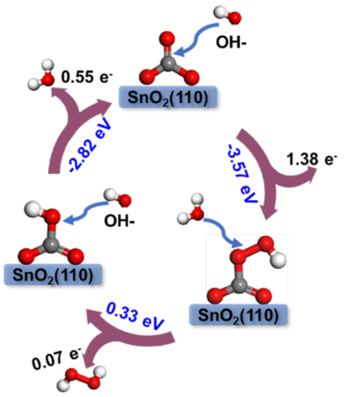

Supplementary Fig. 15 | Calculations of carbonate adsorption-based mechanism on FTO electrode under constant-potential method. The Fermi level of the studied FTO electrode is different from the corresponding actual value under the electrochemical potential, which leads to FTO electrode with non-zero surface charges that affect the chemical reactivity. We tried to study the reactivity under constant potential (U=3.1 V vs RHE pH=12). The calculations using constant potential method suggest that the reaction is unlikely to occur on the surface, due to the unfavorable thermodynamics for the non-electrochemical (i.e. thermal) step: $^*CO_2\text{-}OOH + H_2O \rightarrow {}^*HCO_3 + H_2O_2$. In contrast, the reaction in solution is thermodynamically favorable for all the steps. Thus, we conclude that the reaction is likely to occur in the solution. For constant potential calculation, we adjust the electron number for every ionic step in order to match the "electrode potential" of the system to the experimental value of 3.1 V vs RHE. For a given structure and electron number, the electron potential Φ with respect to the standard hydrogen electrode (SHE) can be obtained as:

$$\Phi = [E_F - E_{es} - (E_F^{SHE} - E_{es}^{SHE})]/e$$

where E_F is the Fermi level of the system, E_{es} is the electrostatic energy in the middle of implicit solution region, and E_F^{SHE} and E_{es}^{SHE} are the corresponding quantities in SHE. In this work, we use the implicit solvation model as implemented in VASPsol. $E_F^{SHE} - E_{es}^{SHE}$ is benchmarked to be 4.6 eV for VASPsol[7] Note that the net electronic charges are balanced by the ionic charges in the implicit solution, and thus the total system remains charge neutral.

Accuracy of the DFT methodology as applied in our work. The previous study has shown that the balance between accuracy and computational cost in DFT simulations depends on the choice of exchange and correlation functional[8]. Taking the SnO_2 (110) surface as an example, RPBE is more accurate for predicting the adsorption energies of small molecules on transition-metal surfaces and does well for those strong chemisorption systems[9,10] and thus the RPBE is chosen for surface studies. When simulating the reactions occurring in electrolytes, strong chemisorption behavior on transition metal surfaces is not involved. Furthermore, it is known that in water, due to the high polarizability of oxygen, vdW interactions have a significant contribution to the binding. The vdW attraction contributes to strengthening both H-bond and non-H-

bond interactions, and it increases the overall cohesive energy in the liquid. Schmidt et al. have studied the vdW effect on the density of water with the PBE+dispersion (PBE-D) method[11] which includes an interatomic pair potential correction added to the PBE functional. They showed that the density of PBE-D water is very close to the experimental value, and the resulting liquid is also structurally closer to experiments. Therefore, the PBE+D3 is chosen to study the reactions taking place in liquid water.

In the inclusion of exact exchange, some proportion of the local exchange-correlation potential is replaced by Hartree-Fock exact-exchange terms, giving very good results for most systems, especially the electrical properties, such as HSE06. However, it's at least an order of magnitude more expensive than GGA calculations. It is very hard to do even a one-step HSE06 calculation at a suitable level of accuracy in the system we studied (more than 150 atoms). Therefore, we used GGA functionals in the calculation process, which can not only provide relatively accurate results, but also obtain reasonable allocation in terms of time and memory.

The previous study has shown that the cause of the systematic errors is the DFT exchange functionals[12,13] The RPBE functional only differs from the PBE functional in the choice of the mathematical form for the exchange energy enhancement factor. The two functionals, PBE and RPBE, follow the same construction logic and therefore contain the same physics and fulfill the same physical criteria[9] In general, the adsorption energies of small molecules on transition metal surfaces calculated using PBE functional are larger than that of RPBE. However, the specific error analysis needs to refer to the experimental data. We will try to study this aspect in depth in future work.

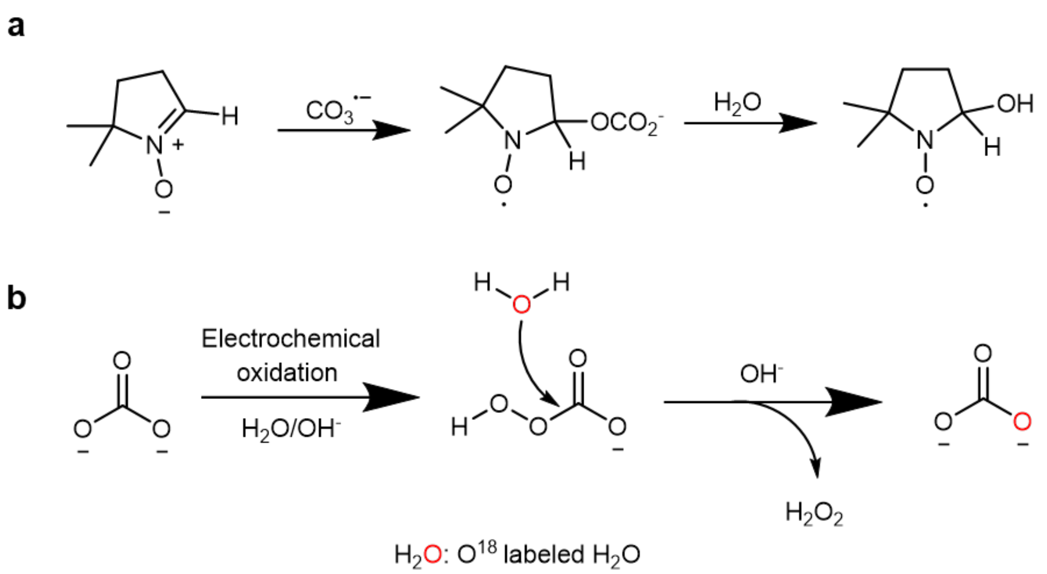

Supplementary Fig. 16 | Experimental mechanistic studies: Reactions in EPR and isotope labeling experiments, as well as control experiment to exclude the carbonate mediator effects on PTFE coated CFP electrode. a, Reaction steps of carbonate radical and DMPO. DMPO spin trap will first react with carbonate radical, and then hydrolyze to form DMPO$^{\bullet}$−OH adduct, which can be detected by EPR spectrum[14]. **b,** The schematic of oxygen exchange between carbonate and water due to the formation of HCO_4^- intermediate. The chemical bond reconfiguration between the percarbonate intermediate and H_2O will cause oxygen exchange in carbonate, leading to the increased abundance of ^{18}O isotope.

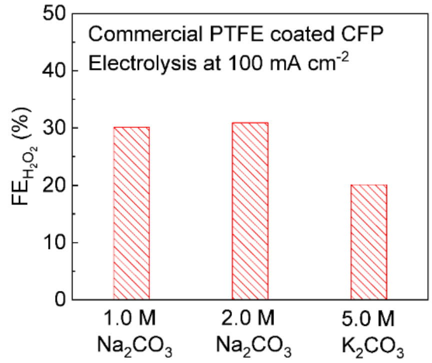

Supplementary Fig. 17 | Control experiment to exclude the carbonate mediator effects on PTFE coated CFP electrode. H_2O_2 FEs of PTFE coated carbon fiber paper (CFP) electrode in different concentrations of carbonate solutions. In our previous work[15], we use hydrophobic PTFE coated carbon fiber paper as a model electrocatalysts to promote water oxidation to H_2O_2 through the local oxygen confinement concept. We noticed that the reaction mechanism of $2e^-$-WOR on this PTFE-coated carbon electrode could be significantly different from that on FTO in this current work, although both tests were operated in carbonate solutions. First, based on isotope experiments in our previous work, we did not observe obvious oxygen exchanges between water and carbonate. Second, we tested the H_2O_2 FEs of PTFE-CFP electrode (commercial hydrophobic carbon paper from Fuel Cell Store, 60wt.% PTFE loading) as a function of carbonate concentrations under a fixed WOR current of 100 mA cm^{-2}, which presented a completely different trend compared to our current study. Lastly, the WOR potentials we tested on PTFE-CFP electrode are ~ 1 V lower than that of FTO, with onset potentials even lower than the theoretical potential of carbonate oxidation to carbonate radical, which could explain the reason why these two systems do not share the same WOR mechanism.

Supplementary Table 1 | Summary of electrochemical 2e⁻-WOR performances.

Catalytic system	Max. FE (%)	Max. $j_{H_2O_2}$ (mA cm^{-2})	Stability (h) @ applied current density	Ref.
FTO/5.0 M K_2CO_3	87	1308	250 h @ 150 mA cm^{-2}	This work
FTO/2.0 M Na_2CO_3	71	487	-	This work
CFP-60%/1.0 M Na_2CO_3	66	75.2	7 h @ ~100 mA cm^{-2}	15
$BiVO_4$/1.0 M $NaHCO_3$	70	18.6	-	16
SnO_2/1.0 M $NaHCO_3$	51	4.92	-	16
TiO_2/1.0 M $NaHCO_3$	18	3.28	-	16
WO_3/1.0 M $NaHCO_3$	46	1.83	-	16
$CaSnO_3$/2.0 M $KHCO_3$	76	14.8	12 h @ ~5 mA cm^{-2}	17
ZnO/2.0 M $KHCO_3$	81	~15	72 h @ ~0.7 mA cm^{-2}	18
Carbon/0.1 M NaOH	47	~1	-	19
Tin-porphyrins/ 0.1 M Na_2SO_4	18	0.1	-	20
Boron-doped diamond/ 1.0 M $KHCO_3$	21.5	66.5	-	21

Supplementary Table 2 | pH value of investigated electrolytes.

Electrolyte	pH value
1.0 M $NaHCO_3$	8.4
1.0 M Na_2SO_4	7.0
1.0 M $NaNO_3$	7.0
1.0 M NaOH	14.0
1.0 M $NaClO_4$	7.0
0.1 M Na_2CO_3	11.5
0.5 M Na_2CO_3	11.8
1.0 M Na_2CO_3	12.0
2.0 M Na_2CO_3	12.4
5.0 M K_2CO_3	13.2

Supplementary Table 3 | The average energies of intermediates in an averaging window of 2.0 ps from 5,500 fs to 7,500 fs.

Intermediates	E_{ave} (eV)
$CO_3^{\bullet-}$	-776.62
HCO_4^-	-777.33
$HCO_3^- + H_2O_2$	-777.64
$CO_2 + H_2O_2 + OH^-$	-776.91

Supplementary Table 4 | Inorganic Standard Electrode Potentials[22].

Half-reaction	Electrode Potential
$CO_3^{\bullet-}(aq) + e^- \rightleftharpoons CO_3^{2-}$	+1.57 ± 0.03 V
$OH^\bullet + e^- + H^+ \rightleftharpoons H_2O$	+2.730 ± 0.017 V
$CO_3^{\bullet-}(aq) + e^- \rightleftharpoons CO_3^{2-}$	+2.278 ± 0.03 V *vs*. RHE
$OH^\bullet + e^- + H^+ \rightleftharpoons H_2O$	+3.438 ± 0.017 V *vs*. RHE

Supplementary Table 5 | Total energies (E, eV), zero-point energy (ZPE) corrections, and entropic contributions (TS) of H_2O, H_2, and H_2O_2.

Species	E (eV)	ZPE (eV)	TS (eV)
H_2O(l)	−14.51	0.59	0.67
H_2(g)	−6.98	0.27	0.40
H_2O_2(l)	−18.31	0.71	0.34

Supplementary Note 1 | Inspiration of CO_2-mediated electrochemical water oxidation to hydrogen peroxide.

In biological cells, low concentrations of reactive oxygen species (ROS, including superoxide anion and hydrogen peroxide) function in signal transduction leading to activation of defense responses, such as apoptosis, proliferation, and gene expression. In contrast, high levels lead to oxidative damage of lipids, deoxyribonucleic acid, and proteins.

Due to the increased CO_2 concentration in the atmosphere, researchers found that high concentration CO_2 will cause ROS burst, which means CO_2 will cause the cell to release ROS rapidly, resulting in oxidative damage rapidly. Although the specific mechanism was not clear up to now, there must be some interaction between CO_2 concentration and ROS generation. Inspired by this phenomenon, we hypothesize that CO_2 may also serve as a promoter to increase the H_2O_2 selectivity in the electrochemical water oxidation system.

Supplementary Note 2 | The significance of carbonate-mediated 2e$^-$-WOR and 2e$^-$-ORR.

We first want to discuss a little more about the relationship between 2e$^-$-ORR and 2e$^-$-WOR. As the oxidation state of oxygen in H_2O_2 is −1, which is between the oxidation state of oxygen in O_2 (0) and the oxidation state of oxygen in H_2O (−2), there are two possible ways for electrochemical H_2O_2 generation: one is from two-electron electrochemical oxygen reduction (2e$^-$-ORR) starts from O_2, and another is from two-electron electrochemical water oxidation (2e$^-$-WOR) starts from H_2O. So, they are not competitive with each other, but they are complementary with each other. After optimizing the electrochemical reactor setup, we can realize cogeneration of H_2O_2 from both anode side and cathode side. Like what we demonstrated in Supplementary Fig. 4b, this system can reach a theoretical H_2O_2 FE of 200% (Methods), as two electrons shuttled from anode side to cathode side can maximally produce two H_2O_2 ($2H_2O + O_2 \rightarrow 2H_2O_2$).

Compared with 2e$^-$-ORR, 2e$^-$-WOR does not involve a triple-phase boundary (gas-phase reactant, liquid-phase electrolyte, and solid-phase electrode) like 2e$^-$-ORR, where O_2 needs to be diffused efficiently onto electrode surface, and gas diffusion layer electrode needs to be used. In contrast, the water oxidation reaction is typically not limited by reactant mass diffusions (as the reactant is water, abundant in the electrolyte), which could help deliver extremely high current densities such as over 1.3 A cm^{-2} (Fig. 3c). Due to the O_2 gas diffusion limit, a typical H_2O_2 partial current density through 2e$^-$-ORR was limited to hundreds of mA cm^{-2} [23,24].

We would also like to acknowledge the pros and cons for both 2e$^-$-ORR and 2e$^-$-WOR reactions as they both are worth of extensive studies for their future applications. Compared to significant progress that has been made in 2e$^-$-ORR filed, 2e$^-$-WOR is an under explored area. In this work, we focused on the carbonate promotion effects in 2e$^-$-WOR. Even though the generated H_2O_2 is in carbonate solutions as we need to have the carbonate mediation effect (different from our previous study of solid electrolyte reactor where no salt removal is required[24]), this study represents a very fundamental understanding and technological improvement for 2e$^-$-WOR performance using carbonate mediation. While the downstream product purification process is not the focus of this work, we can think about two ways to utilize the generated H_2O_2. One is to separate it from the sodium carbonate salt (via methods such as extraction or ion separations) so that we can get pure H_2O_2 solutions for use; another way is to obtain sodium percarbonate salts through the treatment method we described in Supplementary Fig. 4, which is also called "solid hydrogen peroxide" since it is an adduct product between sodium carbonate and H_2O_2. Besides, based on this carbonate mediator effect, we can further develop new reactors to make use of this observed phenomenon in the future, such as incorporating carbonate ions into polymer electrolyte

to see if the WOR performance can be translated into a solid electrolyte reactor for direct synthesis of pure H_2O_2 from both $2e^-$-ORR at the cathode and $2e^-$-WOR at the anode. In conclusion, this work serves as a very important foundation for us to better understand its mechanism, and the carbonate mediation process provides a new approach for more advanced cell designs in the future.

References

1. Nørskov, J. K. *et al.* Universality in Heterogeneous Catalysis. *J. Catal.* **209**, 275–278 (2002).
2. Zhao, X. & Liu, Y. Origin of Selective Production of Hydrogen Peroxide by Electrochemical Oxygen Reduction. *J. Am. Chem. Soc.* **143**, 9423–9428 (2021).
3. Zhao, X. & Liu, Y. Unveiling the Active Structure of Single Nickel Atom Catalysis: Critical Roles of Charge Capacity and Hydrogen Bonding. *J. Am. Chem. Soc.* **142**, 5773–5777 (2020).
4. Mäki-Jaskari, M. A. & Rantala, T. T. Theoretical study of oxygen-deficient SnO 2 (110) surfaces. *Phys. Rev. B* **65**, 245428 (2002).
5. Viswanathan, V., Hansen, H. A. & Nørskov, J. K. Selective Electrochemical Generation of Hydrogen Peroxide from Water Oxidation. *J. Phys. Chem. Lett.* **6**, 4224–4228 (2015).
6. Wang, X., Qin, H., Chen, Y. & Hu, J. Sensing Mechanism of SnO2 (110) Surface to CO: Density Functional Theory Calculations. *J. Phys. Chem. C* **118**, 28548–28561 (2014).
7. Mathew, K., Kolluru, V. S. C., Mula, S., Steinmann, S. N. & Hennig, R. G. Implicit self-consistent electrolyte model in plane-wave density-functional theory. *J. Chem. Phys.* **151**, 234101 (2019).
8. Dick, S. & Fernandez-Serra, M. Machine learning accurate exchange and correlation functionals of the electronic density. *Nat. Commun.* **11**, 3509 (2020).
9. Hammer, B., Hansen, L. B. & Nørskov, J. K. Improved adsorption energetics within density-functional theory using revised Perdew-Burke-Ernzerhof functionals. *Phys. Rev. B* **59**, 7413 (1999).
10. Hensley, A. J. R. *et al.* DFT-Based Method for More Accurate Adsorption Energies: An Adaptive Sum of Energies from RPBE and vdW Density Functionals. *J. Phys. Chem. C* **121**, 4937–4945 (2017).
11. Schmidt, J. *et al.* Isobaric−Isothermal Molecular Dynamics Simulations Utilizing Density Functional Theory: An Assessment of the Structure and Density of Water at Near-Ambient Conditions. *J. Phys. Chem. B* **113**, 11959–11964 (2009).
12. Garza, A. J., Bell, A. T. & Head-Gordon, M. Nonempirical Meta-Generalized Gradient Approximations for Modeling Chemisorption at Metal Surfaces. *J. Chem. Theory Comput.* **14**, 3083–3090 (2018).
13. Brittain, D. R. B. *et al.* The role of exchange in systematic DFT errors for some organic reactions. *Phys. Chem. Chem. Phys.* **11**, 1138–1142 (2009).
14. Medinas, D. B., Cerchiaro, G., Trindade, D. F. & Augusto, O. The carbonate radical and related oxidants derived from bicarbonate buffer. *IUBMB Life* **59**, 255–262 (2007).
15. Xia, C. *et al.* Confined local oxygen gas promotes electrochemical water oxidation to hydrogen peroxide. *Nat. Catal.* **3**, 125–134 (2020).
16. Shi, X. *et al.* Understanding activity trends in electrochemical water oxidation to form hydrogen peroxide. *Nat. Commun.* **8**, 701 (2017).

17. Park, S. Y. *et al.* CaSnO3: An Electrocatalyst for Two-Electron Water Oxidation Reaction to Form H2O2. *ACS Energy Lett.* **4**, 352–357 (2019).
18. Kelly, S. R. *et al.* ZnO As an Active and Selective Catalyst for Electrochemical Water Oxidation to Hydrogen Peroxide. *ACS Catal.* **9**, 4593–4599 (2019).
19. Ando, Y. Proposal for a new system for simultaneous production of hydrogen and hydrogen peroxide by water electrolysis. *Int. J. Hydrog. Energy* **29**, 1349–1354 (2004).
20. Ohsaki, Y. *et al.* Two-electron oxidation of water to form hydrogen peroxide initiated by one-electron oxidation of Tin (IV)-porphyrins. *J. Photochem. Photobiol. Chem.* **401**, 112732 (2020).
21. Mavrikis, S., Göltz, M., Rosiwal, S., Wang, L. & Ponce de León, C. Boron-Doped Diamond Electrocatalyst for Enhanced Anodic H_2O_2 Production. *ACS Appl. Energy Mater.* **3**, 3169–3173 (2020).
22. Armstrong, D. A. *et al.* Standard electrode potentials involving radicals in aqueous solution: inorganic radicals. *Bioinorg. React. Mech.* **9**, 59–61 (2013).
23. Perry, S. C. Electrochemical synthesis of hydrogen peroxide from water and oxygen. *Nat. Rev. Chem.* 17 (2019).
24. Xia, C., Xia, Y., Zhu, P., Fan, L. & Wang, H. Direct electrosynthesis of pure aqueous H_2O_2 solutions up to 20% by weight using a solid electrolyte. *Science* **366**, 226–231 (2019).

The structure of CO_3 adsorption on SnO_2(110) surface.

```
Sn48O99C1
   1.0000000000000000
    12.9239999999999995    0.0000000000000000    0.0000000000000000
     0.0000000000000008   13.5635999999999992    0.0000000000000000
     0.0000000000000015    0.0000000000000015   24.4095000000000013
   Sn   O    C
   48   99   1
Direct
  0.1250000000000000  0.0241300000000000  0.0538300000000000
  0.1250800000000000  0.2737500000000000  0.1925000000000000
  0.1254300000000000  0.0242200000000000  0.3232400000000000
  0.0000000000000000  0.2741300000000000  0.0538300000000000
  0.0001100000000000  0.0239700000000000  0.1886800000000000
  0.0002900000000000  0.2742300000000000  0.3328400000000000
  0.3750000000000000  0.0241300000000000  0.0538300000000000
  0.3749500000000000  0.2737000000000000  0.1924900000000000
  0.3753900000000000  0.0241400000000000  0.3230000000000000
  0.2500000000000000  0.2741300000000000  0.0538300000000000
  0.2500000000000000  0.0239500000000000  0.1883400000000000
  0.2500200000000000  0.2748700000000000  0.3325200000000000
  0.6250000000000000  0.0241300000000000  0.0538300000000000
  0.6252400000000000  0.2736900000000000  0.1924800000000000
  0.6251200000000000  0.0241700000000000  0.3230000000000000
  0.5000000000000000  0.2741300000000000  0.0538300000000000
  0.5001000000000000  0.0239500000000000  0.1884600000000000
  0.5002600000000000  0.2756700000000000  0.3309900000000000
  0.8750000000000000  0.0241300000000000  0.0538300000000000
  0.8751100000000001  0.2737500000000000  0.1925000000000000
  0.8750500000000000  0.0242200000000000  0.3232300000000000
  0.7500000000000000  0.2741300000000000  0.0538300000000000
  0.7502200000000000  0.0239600000000000  0.1883200000000000
  0.7505500000000001  0.2749100000000000  0.3324900000000000
  0.1250000000000000  0.5241200000000000  0.0538300000000000
  0.1251000000000000  0.7740400000000000  0.1925000000000000
  0.1258100000000000  0.5241700000000000  0.3221100000000000
  0.0000000000000000  0.7741200000000000  0.0538300000000000
  0.0001500000000000  0.5239400000000000  0.1878700000000000
  0.0003000000000000  0.7740200000000000  0.3328500000000000
  0.3750000000000000  0.5241200000000000  0.0538300000000000
  0.3749600000000000  0.7741200000000000  0.1924800000000000
  0.3778100000000000  0.5240000000000000  0.3268900000000000
  0.2500000000000000  0.7741200000000000  0.0538300000000000
```

0.2513100000000000	0.5239800000000000	0.1887300000000000
0.2500300000000000	0.7733200000000000	0.3325200000000000
0.6250000000000000	0.5241200000000000	0.0538300000000000
0.6252799999999999	0.7741300000000000	0.1924700000000000
0.6228700000000000	0.5240700000000000	0.3267900000000000
0.5000000000000000	0.7741200000000000	0.0538300000000000
0.5001200000000000	0.5239700000000000	0.1928500000000000
0.5003000000000000	0.7724700000000000	0.3309800000000000
0.8750000000000000	0.5241200000000000	0.0538300000000000
0.8751500000000000	0.7740500000000000	0.1925000000000000
0.8748400000000000	0.5242400000000000	0.3220700000000000
0.7500000000000000	0.7741200000000000	0.0538300000000000
0.7489600000000000	0.5239800000000000	0.1887000000000000
0.7505400000000000	0.7733100000000001	0.3325000000000000
0.1250000000000000	0.2741300000000000	0.0000000000000000
0.1247300000000000	0.0239600000000000	0.1362500000000000
0.1247700000000000	0.2721900000000000	0.2759600000000000
0.1248700000000000	0.2741500000000000	0.1089100000000000
0.1253100000000000	0.0241500000000000	0.2419400000000000
0.1252000000000000	0.2735800000000000	0.3840100000000000
0.0000000000000000	0.1210000000000000	0.0538300000000000
0.0001200000000000	0.3721700000000000	0.1914800000000000
0.0002400000000000	0.1174100000000000	0.3332800000000000
0.0000000000000000	0.4272600000000000	0.0538300000000000
0.0001100000000000	0.1752400000000000	0.1913400000000000
0.0003000000000000	0.4307100000000000	0.3323000000000000
0.3750000000000000	0.2741300000000000	0.0000000000000000
0.3750600000000000	0.0239700000000000	0.1362200000000000
0.3734200000000000	0.2662900000000000	0.2755300000000000
0.3749800000000000	0.2739600000000000	0.1089000000000000
0.3749500000000000	0.0241200000000000	0.2417700000000000
0.3761300000000000	0.2765500000000000	0.3831900000000000
0.2500000000000000	0.1210000000000000	0.0538300000000000
0.2498600000000000	0.3718100000000000	0.1915700000000000
0.2502200000000000	0.1174800000000000	0.3341300000000000
0.2500000000000000	0.4272600000000000	0.0538300000000000
0.2496000000000000	0.1750300000000000	0.1905200000000000
0.2490100000000000	0.4302400000000000	0.3317700000000000
0.6250000000000000	0.2741300000000000	0.0000000000000000
0.6251000000000000	0.0239600000000000	0.1362100000000000
0.6270300000000000	0.2661900000000000	0.2755200000000000
0.6250700000000000	0.2739600000000000	0.1088900000000000
0.6253100000000000	0.0241200000000000	0.2417700000000000
0.6244700000000000	0.2766000000000000	0.3831700000000000

0.5000000000000000	0.1210000000000000	0.0538300000000000
0.5001100000000001	0.3716000000000000	0.1920500000000000
0.5002300000000000	0.1177300000000000	0.3338300000000000
0.5000000000000000	0.4272600000000000	0.0538300000000000
0.5001000000000000	0.1750700000000000	0.1906400000000000
0.5003300000000001	0.4266100000000000	0.3182900000000000
0.8750000000000000	0.2741300000000000	0.0000000000000000
0.8754600000000000	0.0239600000000000	0.1362500000000000
0.8757300000000000	0.2720800000000000	0.2759500000000000
0.8751900000000000	0.2741600000000000	0.1089100000000000
0.8749400000000001	0.0241400000000000	0.2419300000000000
0.8753600000000000	0.2736500000000000	0.3839900000000000
0.7500000000000000	0.1210000000000000	0.0538300000000000
0.7503600000000000	0.3718100000000000	0.1915700000000000
0.7502700000000000	0.1175000000000000	0.3341300000000000
0.7500000000000000	0.4272600000000000	0.0538300000000000
0.7506100000000000	0.1750300000000000	0.1904900000000000
0.7516400000000000	0.4302600000000000	0.3315800000000000
0.1250000000000000	0.7741200000000000	0.0000000000000000
0.1257800000000000	0.5239600000000000	0.1361400000000000
0.1247700000000000	0.7752700000000000	0.2759600000000000
0.1248700000000000	0.7741000000000000	0.1089000000000000
0.1244900000000000	0.5241900000000000	0.2409800000000000
0.1252100000000000	0.7748600000000000	0.3840200000000000
0.0000000000000000	0.6209900000000000	0.0538300000000000
0.0001300000000000	0.8726800000000000	0.1915300000000000
0.0003300000000000	0.6175000000000000	0.3325900000000000
0.0000000000000000	0.9272600000000000	0.0538300000000000
0.0001300000000000	0.6757200000000000	0.1912900000000000
0.0002400000000000	0.9308300000000000	0.3330600000000000
0.3750000000000000	0.7741200000000000	0.0000000000000000
0.3770600000000000	0.5239900000000000	0.1368900000000000
0.3734100000000000	0.7813800000000000	0.2755200000000000
0.3749800000000000	0.7742700000000000	0.1089000000000000
0.3714900000000000	0.5241400000000001	0.2432200000000000
0.3761300000000000	0.7718200000000000	0.3831900000000000
0.2500000000000000	0.6209900000000000	0.0538300000000000
0.2495900000000000	0.8728700000000000	0.1906700000000000
0.2490800000000000	0.6178600000000000	0.3320400000000000
0.2500000000000000	0.9272600000000000	0.0538300000000000
0.2498700000000000	0.6761100000000000	0.1914100000000000
0.2501800000000000	0.9307100000000000	0.3338800000000000
0.6250000000000000	0.7741200000000000	0.0000000000000000
0.6231200000000000	0.5239800000000000	0.1368800000000000

0.6270600000000000	0.7814200000000000	0.2755100000000000
0.6250700000000000	0.7742800000000000	0.1088900000000000
0.6288100000000000	0.5241800000000000	0.2431600000000000
0.6244700000000000	0.7717500000000000	0.3831700000000000
0.5000000000000000	0.6209900000000000	0.0538300000000000
0.5001100000000001	0.8728300000000000	0.1907700000000000
0.5002799999999999	0.6214600000000000	0.3184400000000000
0.5000000000000000	0.9272600000000000	0.0538300000000000
0.5001200000000000	0.6763100000000000	0.1919500000000000
0.5002600000000000	0.9304400000000000	0.3336500000000000
0.8750000000000000	0.7741200000000000	0.0000000000000000
0.8744499999999999	0.5239500000000000	0.1361300000000000
0.8757400000000000	0.7753600000000000	0.2759500000000000
0.8751900000000000	0.7741100000000000	0.1089000000000000
0.8758500000000000	0.5242000000000000	0.2409500000000000
0.8753600000000000	0.7748000000000000	0.3840000000000000
0.7500000000000000	0.6209900000000000	0.0538300000000000
0.7506500000000000	0.8728800000000000	0.1906400000000000
0.7515700000000000	0.6179100000000000	0.3318500000000000
0.7500000000000000	0.9272600000000000	0.0538300000000000
0.7503700000000000	0.6761200000000001	0.1914000000000000
0.7503000000000000	0.9307100000000000	0.3338900000000000
0.5913400000000000	0.5238300000000000	0.4114900000000000
0.5010800000000000	0.5213600000000000	0.4885400000000000
0.4102900000000000	0.5237000000000001	0.4116200000000000
0.5008700000000000	0.5229100000000000	0.4365900000000000

The structure of $CO_3^{\bullet-}$ with 50 H_2O molecules employed in the AIMD simulations.

O54H101C1
1.0000000000000000

12.0000000000000000	0.0000000000000000	0.0000000000000000
0.0000000000000007	12.0000000000000000	0.0000000000000000
0.0000000000000007	0.0000000000000007	12.0000000000000000

O H C
54 101 1
Direct

0.1212400000000000	0.8345399999999999	0.3205800000000000
0.7836700000000000	0.5485500000000000	0.0564500000000000
0.7299800000000000	-0.0142800000000000	0.4894100000000000
0.5247300000000000	0.8953300000000000	0.3864900000000000
0.2473500000000000	0.5482200000000000	0.5319600000000000

0.3288900000000000	0.2085300000000000	0.8423300000000000
0.6642800000000000	0.7449000000000000	0.2821600000000000
0.8741800000000000	0.3662100000000000	0.3347000000000000
0.4723700000000000	0.0998500000000000	0.3198500000000000
0.6742600000000000	0.4416300000000000	0.9004400000000000
0.4419600000000000	0.2174000000000000	0.5475500000000000
0.7647300000000000	0.1549200000000000	0.8343900000000000
0.1492000000000000	0.6203700000000000	0.7290900000000000
0.6696100000000000	0.7238100000000000	0.6603100000000000
0.4467900000000000	0.7221100000000000	0.6425300000000000
0.3230100000000000	0.7114100000000000	0.3625700000000000
0.7718600000000000	0.8378800000000000	0.8315800000000000
0.2710700000000000	0.1181600000000000	0.6531100000000000
-0.0993600000000000	-0.0592600000000000	0.6575800000000001
0.8776100000000000	0.3003700000000000	0.5598900000000000
0.6740200000000000	0.1636700000000000	0.6116700000000000
0.9739600000000000	0.4636700000000000	0.9497600000000000
0.3675100000000000	0.6362800000000000	0.8142000000000000
0.6020100000000000	0.9188400000000000	0.9979500000000000
0.0755000000000000	0.0443300000000000	0.3568300000000000
0.0444300000000000	0.5273500000000000	0.2828100000000000
0.0471000000000000	0.1145100000000000	0.5725100000000000
0.7000300000000000	0.4468800000000000	0.2411400000000000
0.5456100000000000	0.1663300000000000	0.9794600000000000
0.0984300000000000	0.8202199999999999	0.0829100000000000
-0.0155300000000000	0.4653400000000000	0.7092100000000000
0.9286700000000000	0.2387200000000000	0.9645899999999999
0.2891000000000000	0.8815700000000000	0.6717800000000000
0.2711000000000000	0.9691600000000000	0.0560200000000000
0.0841600000000000	0.7958600000000000	0.6011700000000000
0.7440099999999999	0.8987500000000000	0.1627300000000000
0.4087600000000000	0.8004900000000000	-0.0050900000000000
0.1704100000000000	0.1841000000000000	0.9721800000000000
0.4128200000000000	0.4085600000000000	0.8874000000000000
0.1350100000000000	0.5838000000000000	0.0908900000000000
0.1817100000000000	0.3483500000000000	0.1832500000000000
0.8549900000000000	0.0524800000000000	0.2983100000000000
0.9450600000000000	0.7602400000000000	0.4441800000000000
0.3514300000000000	0.6168500000000000	0.1421800000000000
0.5448200000000000	0.2826700000000000	0.1807100000000000
0.8624400000000000	0.7679500000000000	0.0380500000000000
0.7580500000000000	0.5181200000000000	0.7013200000000001
0.2573100000000000	0.1292500000000000	0.2264200000000000
0.3828700000000000	0.4001000000000000	0.1054100000000000

0.8338700000000000	0.2369600000000000	0.1650600000000000
0.6313200000000000	0.4148600000000000	0.5303900000000000
0.5441300000000000	0.5497800000000000	0.4307700000000000
0.4361600000000000	0.4260800000000000	0.5157000000000000
0.7322500000000000	0.7125700000000000	0.4466900000000000
0.1041500000000000	0.8307500000000000	0.2382900000000000
0.1074200000000000	0.9116700000000000	0.3452000000000000
0.7552600000000000	0.5203700000000000	0.1293900000000000
0.7275700000000001	0.5133500000000000	1.0023599999999999
0.6635300000000000	-0.0498700000000000	0.4671900000000000
0.7086100000000000	0.0508500000000000	0.5390000000000000
0.7642400000000000	0.4181400000000000	0.2893400000000000
0.5116500000000000	-0.0325300000000000	0.3441800000000000
0.4525400000000000	0.8580300000000000	0.3772600000000000
0.4842200000000000	0.1751100000000000	0.9288700000000000
0.2175600000000000	0.5779500000000000	0.6051299999999999
0.3012800000000000	0.4899800000000000	0.5420100000000000
0.2982500000000000	0.1705400000000000	0.7707800000000000
0.3568000000000000	0.2869400000000000	0.8381800000000000
0.7151200000000000	0.7931900000000000	0.2401700000000000
0.6075400000000000	0.7947800000000000	0.3175300000000000
0.8841599999999999	0.3066400000000000	0.2738200000000000
-0.0729800000000000	0.4270500000000000	0.3253900000000000
0.5053200000000000	0.1689900000000000	0.2834100000000000
0.3992900000000000	0.1021200000000000	0.2870600000000000
0.0766400000000000	0.5554000000000000	0.2108600000000000
0.0580400000000000	0.5787700000000000	0.3436800000000000
0.5940900000000000	0.4399500000000000	0.8954100000000000
0.6961900000000000	0.4785900000000000	0.8282800000000000
0.4441800000000000	0.2973600000000000	0.5302800000000000
0.4489700000000000	0.1863000000000000	0.4736500000000000
0.1540700000000000	0.8682299999999999	0.0495100000000000
0.0025600000000000	0.0581800000000000	0.6136100000000000
0.7019500000000000	0.1827100000000000	0.8802900000000000
0.2145200000000000	0.6274999999999999	0.7803900000000000
0.1051700000000000	0.5527400000000000	0.7267600000000000
0.6942900000000000	0.6516900000000000	0.6898700000000000
0.6994300000000000	0.7250799999999999	0.5811900000000000
0.4219100000000000	0.6792899999999999	0.5761200000000000
0.5304600000000000	0.7252500000000000	0.6323800000000001
0.2589700000000000	0.7608900000000000	0.3516800000000000
0.3005900000000000	0.6574000000000000	0.4247100000000000
0.8245300000000000	0.8953100000000001	0.7987300000000001
0.7377200000000000	0.8025700000000000	0.7653100000000000

0.2906100000000000	0.0330100000000000	0.6422400000000000
-0.0775400000000000	0.2315100000000000	0.5552100000000000
0.8715700000000000	0.3237500000000000	0.4773500000000000
0.7234900000000000	0.2282100000000000	0.5815500000000000
0.5930400000000000	0.1845800000000000	0.6020100000000000
-0.0481500000000000	0.3850400000000000	-0.0492400000000000
0.9082400000000000	0.4871100000000000	0.9957700000000000
0.3787500000000000	0.6990100000000000	0.8694400000000000
0.4055400000000000	0.6588500000000000	0.7461500000000000
0.8402300000000000	0.9303300000000000	0.6023500000000001
0.5346300000000000	0.8671700000000000	-0.0074300000000000
0.6459900000000000	0.9167100000000000	0.9320900000000000
-0.0408200000000000	0.8840100000000000	0.6407700000000000
0.9953600000000000	0.0351900000000000	0.3307200000000000
0.0614300000000000	0.0770600000000000	0.4343900000000000
0.1222800000000000	0.1289900000000000	0.6055800000000000
0.6594600000000000	0.4991300000000000	0.2832300000000000
0.5553900000000001	0.0849600000000000	0.9883500000000000
1.0232100000000000	0.8107200000000000	0.0452300000000000
0.7456700000000001	0.1537500000000000	0.7568900000000000
0.3221300000000000	0.1662800000000000	0.6066400000000000
-0.0481200000000000	0.4043100000000000	0.6617800000000000
-0.0188600000000000	0.4358200000000000	0.7860000000000000
0.8807700000000001	0.1948200000000000	0.9162500000000000
1.0080100000000001	0.2157500000000000	0.9492600000000000
0.3481800000000000	-0.1745500000000000	0.6607900000000000
0.2211200000000000	0.8550700000000000	0.6348300000000000
0.2502300000000000	1.0076300000000000	0.9847900000000001
0.2587700000000000	1.0266599999999999	0.1136000000000000
0.0907800000000000	0.7312600000000000	0.6510300000000000
0.0333800000000000	0.7736800000000000	0.5424099999999999
0.7950000000000000	0.8614700000000000	0.1067700000000000
0.6754900000000000	0.9179600000000000	0.1127800000000000
0.4043100000000000	0.7408100000000000	0.0543300000000000
0.3606200000000000	0.8649500000000000	0.0182600000000000
0.1801000000000000	0.2383300000000000	1.0296200000000000
0.2255100000000000	0.1992600000000000	0.9089400000000000
0.4211100000000000	0.4138800000000000	0.9738800000000000
0.3968500000000000	0.4878200000000000	0.8713100000000000
0.1176900000000000	0.6631500000000000	0.0739100000000000
0.0933800000000000	0.5415900000000000	0.0327100000000000
0.1372900000000000	0.4111700000000000	0.2131500000000000
0.2550600000000000	0.3833600000000000	0.1789100000000000
0.8221700000000000	0.9921300000000000	0.2473100000000000

0.8098800000000000	0.0373900000000000	0.3678100000000000
0.8622200000000000	0.7575000000000000	0.4283000000000000
0.9941500000000000	0.7764000000000000	0.3814100000000000
0.2823000000000000	0.6016100000000000	0.1012000000000000
0.3300100000000000	0.6396400000000000	0.2181800000000000
0.6153600000000000	0.3333400000000000	0.1941000000000000
0.5535099999999999	0.2455700000000000	0.1015000000000000
0.8358000000000000	0.7796700000000000	0.9622400000000000
0.8378600000000000	0.6883600000000000	0.0479500000000000
0.7288800000000000	0.4759000000000000	0.6401300000000000
0.8427800000000000	0.5251100000000000	0.6962900000000000
0.2446300000000000	0.2076800000000000	0.2198800000000000
0.1929200000000000	0.1046200000000000	0.2718600000000000
0.3835200000000000	0.4816600000000000	0.1176100000000000
0.4479400000000000	0.3724100000000000	0.1459500000000000
0.8905700000000000	0.2341300000000000	0.1009200000000000
0.8364100000000000	0.1620100000000000	0.2028400000000000
0.6793700000000000	0.6535300000000001	0.4260400000000000
0.5349300000000000	0.4644200000000000	0.4938500000000000

The structure of HCO_4^- with 50 H_2O molecules employed in the AIMD simulations.

```
O54H101C1
   1.0000000000000000
  12.0000000000000000    0.0000000000000000    0.0000000000000000
   0.0000000000000007   12.0000000000000000    0.0000000000000000
   0.0000000000000007    0.0000000000000007   12.0000000000000000
  O    H    C
  54   101  1
Direct
   0.1575100000000000    0.6721900000000000    0.4156200000000000
   0.4366000000000000    0.5637100000000000    0.1995200000000000
   0.6487500000000000    1.0000000000000000    0.8335300000000000
   0.4005300000000000    0.0326900000000000    0.6048000000000000
   0.6635600000000000    0.6862500000000000    0.9076400000000000
   0.3031600000000000    0.3308900000000000    0.7382700000000000
   0.6220599999999999    1.0272800000000000    0.3005000000000000
   0.7933100000000000    0.2648200000000000    0.2768000000000000
   0.5237300000000000    0.2911600000000000    0.3752300000000000
   0.5629300000000000    0.8558700000000000    0.9916700000000001
   0.5882100000000000    0.3867400000000000    0.1987900000000000
   0.9364700000000000    0.2217400000000000    0.7846800000000000
   0.0333800000000000    0.5466200000000000    0.7137100000000000
```

0.6857600000000000	0.7985500000000000	0.7122900000000000
0.3592400000000000	0.3990000000000000	0.5203100000000001
0.2450600000000000	0.4930900000000000	0.3435900000000000
0.8094400000000000	0.7443700000000000	0.2822700000000000
0.1233000000000000	0.7427700000000000	0.6219300000000000
0.5755600000000000	0.1459600000000000	0.6806200000000000
0.1027900000000000	0.3814700000000000	0.8382400000000000
0.9244599999999999	0.2752100000000000	0.5836700000000000
0.7896800000000000	0.3118900000000000	0.9297000000000000
0.6381100000000000	0.4604800000000000	0.8404199999999999
0.4483300000000000	0.1380500000000000	0.2042800000000000
0.1729800000000000	0.8155600000000000	0.2415900000000000
0.9212000000000000	0.4419300000000000	0.2086400000000000
0.7075300000000000	0.1389200000000000	0.5076900000000000
0.8808400000000000	0.5011800000000000	-0.0137600000000000
0.5064800000000000	0.2820400000000000	0.8327500000000000
0.8771500000000000	1.0227700000000000	0.8176300000000000
0.0168700000000000	0.6654200000000000	0.9299700000000000
0.8729900000000000	-0.0294200000000000	0.0536800000000000
0.3858200000000000	0.6793900000000000	0.7842700000000000
0.2656000000000000	0.9385900000000000	0.9537200000000000
0.8400900000000000	0.4890100000000000	0.5979800000000000
0.9348400000000000	0.0295100000000000	0.4727500000000000
0.3878200000000000	0.9010100000000000	0.1326900000000000
0.2672800000000000	0.8654800000000000	0.7437400000000000
0.1286900000000000	0.3353600000000000	0.2294100000000000
0.4143900000000000	0.6618500000000000	0.9987800000000000
0.1313100000000000	0.1117100000000000	0.9667200000000000
0.0160300000000000	0.0060900000000000	0.2375000000000000
0.9239000000000001	0.8660500000000000	0.6473100000000001
0.2025400000000000	0.7588300000000000	0.0162800000000000
0.4503900000000000	0.2785300000000000	0.0372100000000000
0.8056500000000000	0.7610200000000000	1.0543300000000000
0.9344000000000000	0.5798100000000000	0.4065800000000000
0.5178800000000000	0.8593000000000000	0.4303700000000000
0.2272200000000000	0.2973800000000000	0.0263600000000000
0.7297600000000000	0.1495100000000000	0.0729000000000000
0.5112400000000000	0.6683100000000000	0.6075300000000000
0.5628400000000000	0.5726599999999999	0.4571000000000000
0.6089900000000000	0.5013000000000000	0.6179700000000000
0.4844600000000000	0.6504400000000000	0.4014900000000000
0.1447900000000000	0.7038900000000000	0.4907300000000000
0.1584900000000000	0.7286200000000000	0.3557600000000000
0.4967900000000000	0.5034000000000000	0.1828100000000000

0.4630500000000000	0.6106400000000000	0.2587700000000000
0.6142800000000000	0.0506100000000000	0.7792200000000000
0.7325400000000000	1.0198400000000001	0.8352500000000000
0.8817700000000001	0.4901300000000000	0.0657700000000000
0.3711000000000000	0.0875600000000000	0.5508100000000000
0.4628700000000000	0.0753400000000000	0.6407600000000000
0.4287600000000000	0.2882100000000000	0.8072000000000000
0.6856200000000000	0.7179100000000000	0.8322600000000000
0.6078500000000000	0.7454100000000000	0.9387000000000000
0.3172400000000000	0.3561100000000000	0.6606500000000000
0.2283600000000000	0.3509700000000000	0.7633799999999999
0.6843500000000000	1.0501499999999999	0.2522500000000000
0.5619000000000000	1.0817500000000000	0.2938000000000000
0.8601000000000000	0.3033600000000000	0.2602400000000000
0.7661900000000000	0.3000200000000000	0.3458500000000000
0.4797400000000000	0.2368400000000000	0.3346500000000000
0.4702600000000000	0.3268700000000000	0.4261700000000000
1.0021199999999999	0.4256600000000000	0.2050100000000000
0.9189100000000000	0.4949900000000000	0.2751600000000000
0.5294800000000000	0.8790200000000000	1.0605599999999999
0.6021700000000000	0.9146200000000000	0.9517300000000000
0.6637100000000000	0.4104100000000000	0.1742600000000000
0.5832100000000000	0.3499300000000000	0.2774700000000000
0.9154900000000000	1.0982900000000000	0.8042100000000000
0.7863800000000000	0.1139400000000000	0.5040400000000000
0.8867100000000000	0.2566300000000000	0.8447200000000000
0.9664600000000000	0.5277100000000000	0.6737100000000000
0.0856600000000000	0.6019300000000000	0.6739300000000000
0.6248100000000000	0.7761700000000000	0.6630500000000000
0.6576800000000000	0.8737700000000000	0.7415800000000000
0.3128900000000000	0.4301900000000000	0.4595700000000000
0.4178700000000000	0.4554100000000000	0.5311800000000000
0.3069500000000000	0.5143500000000000	0.2996800000000000
0.2127400000000000	0.5638400000000000	0.3817100000000000
0.7938900000000000	0.7355600000000000	0.2031700000000000
0.8241500000000000	0.8229500000000000	0.2921700000000000
0.0524500000000000	0.7884400000000000	0.6474000000000000
0.0985200000000000	0.4454600000000000	0.7866300000000001
0.0439200000000000	0.3279700000000000	0.8059200000000000
0.8879200000000000	0.3508400000000000	0.5899400000000000
0.9140600000000000	0.2523400000000000	0.5066000000000001
0.8322700000000000	0.3791800000000000	0.9523000000000000
0.7240100000000000	0.3524600000000000	0.8941600000000000
0.6536200000000000	0.5304600000000000	0.8808900000000000

0.6273600000000000	0.4863800000000000	0.7617699999999999
0.6220500000000000	0.1595300000000000	0.6159200000000000
0.4264000000000000	0.0589100000000000	0.1868700000000000
0.4432800000000000	0.1765500000000000	0.1359900000000000
0.5682900000000000	0.2167000000000000	0.7249400000000000
0.1826400000000000	0.7935700000000000	0.1625900000000000
0.1052000000000000	0.8590500000000000	0.2382700000000000
0.6648500000000001	0.0987800000000000	0.4491600000000000
0.9406800000000000	0.5553399999999999	-0.0383500000000000
0.5452700000000000	0.3533500000000000	0.8446900000000001
0.8963200000000000	1.0041800000000001	0.8968200000000000
0.9064500000000000	0.2192200000000000	0.7030300000000000
0.1860400000000000	0.7745200000000000	0.6703300000000000
0.0144900000000000	0.6276000000000000	0.8561000000000000
0.0876200000000000	0.6968700000000000	0.9539700000000000
0.8348300000000000	-0.1036100000000000	0.0661800000000000
0.8253200000000001	0.0418500000000000	0.0672200000000000
0.4458200000000000	0.6759700000000000	0.7268700000000000
0.4053200000000000	0.6419000000000000	0.8551200000000000
0.2755500000000000	0.9241700000000000	0.8690200000000000
0.2070900000000000	0.9995400000000000	0.9537600000000001
0.8599800000000000	0.5212300000000000	0.5242800000000000
0.7542700000000000	0.5009400000000001	0.6034300000000000
0.9430500000000001	-0.0372600000000000	0.5238000000000000
0.9364500000000000	-0.0032700000000000	0.3943400000000000
0.3408700000000000	0.9088900000000000	0.0599100000000000
0.3345300000000000	0.8721400000000000	0.1910200000000000
0.3210400000000000	0.7988400000000000	0.7537400000000000
0.3033300000000000	0.9178900000000000	0.6905300000000000
0.1743000000000000	0.3927600000000000	0.2763700000000000
0.1708300000000000	0.3221000000000000	0.1597600000000000
0.4501300000000000	0.7314400000000000	0.9849900000000000
0.4312500000000000	0.6307400000000000	0.0718700000000000
0.1755500000000000	0.1778700000000000	-0.0165800000000000
0.0774500000000000	0.1331700000000000	0.9105500000000000
0.0584100000000000	0.0753600000000000	0.2443800000000000
0.9667900000000000	0.0151900000000000	0.1690600000000000
0.8533700000000000	0.8245700000000000	0.6546300000000000
0.9227600000000000	0.9265700000000000	0.7062800000000000
0.2749400000000000	0.7172300000000000	0.0104500000000000
0.2231300000000000	0.8356900000000000	0.9928900000000001
0.4784700000000000	0.2820500000000000	-0.0427400000000000
0.4994700000000000	0.3174000000000000	0.0899600000000000
0.7594500000000000	0.7350300000000000	0.9931400000000000

```
0.8838400000000000    0.7398600000000000    1.0384000000000000
0.8953900000000000    0.6435200000000000    0.3783400000000000
1.0116099999999999    0.6073700000000000    0.4109500000000000
0.4928200000000000    0.8936100000000000    0.5002700000000000
0.5670300000000000    0.9202399999999999    0.3917100000000000
0.1889800000000000    0.3483500000000000    0.9751900000000000
0.3118900000000000    0.3060500000000000    0.0218300000000000
0.7623300000000000    0.2030100000000000    0.0203900000000000
0.7504999999999999    0.1820600000000000    0.1462800000000000
0.5186500000000001    0.7291000000000000    0.4091700000000000
0.5592800000000000    0.5845300000000000    0.5724500000000000
```

The structure of HCO_3^- + H_2O_2 with 49 H_2O molecules employed in the AIMD simulations.

```
O54H101C1
   1.0000000000000000
  12.0000000000000000    0.0000000000000000    0.0000000000000000
   0.0000000000000007   12.0000000000000000    0.0000000000000000
   0.0000000000000007    0.0000000000000007   12.0000000000000000
  O    H    C
  54   101  1
Direct
   0.3466400000000000    0.9069000000000000    0.3157600000000000
   0.0054700000000000    0.5450800000000000    0.1656600000000000
   0.5656400000000000    0.0483800000000000    0.6348500000000000
   0.3805000000000000    0.1543300000000000    0.5575500000000000
   0.6176700000000001    0.7367600000000000    0.1743800000000000
   0.4725200000000000    0.2195200000000000    0.3496900000000000
   0.6239200000000000    0.9232300000000000    0.4484200000000000
   1.0437200000000000    0.2120600000000000    0.2712100000000000
   0.1985400000000000    0.1872500000000000    0.1245900000000000
   0.6167899999999999    0.9477400000000000    0.0795600000000000
   0.0245900000000000    0.3490500000000000    0.7644600000000000
   0.4105500000000000    0.3080500000000000    1.1412500000000001
   0.2812000000000000    0.5940900000000000    0.9373500000000000
   0.9357400000000000    0.8934700000000000    0.5416800000000001
   0.6442700000000000    0.7062700000000000    0.7637200000000000
   0.5626700000000000    0.6850000000000001    0.4474300000000000
   0.8527300000000000    0.9354200000000000    0.1679700000000000
   0.1701600000000000    1.0254700000000001    0.5453600000000000
  -0.1226700000000000    0.0133700000000000    0.7864500000000000
   0.1178100000000000    0.4252400000000000    0.9705000000000000
```

0.8919800000000000	0.1811800000000000	0.5291100000000000
0.5246900000000000	0.1667800000000000	-0.0041900000000000
0.7246100000000000	0.6974600000000000	-0.0335200000000000
0.1487300000000000	-0.2120500000000000	-0.0264500000000000
0.1477800000000000	0.9957400000000000	0.3336900000000000
0.3279500000000000	0.4937800000000000	0.7275100000000000
0.0410100000000000	0.1303400000000000	0.6977300000000000
0.8722400000000000	0.5437500000000000	-0.0263600000000000
0.5041099999999999	0.3720000000000000	0.8879200000000000
0.8067000000000000	0.3561700000000000	0.8356400000000000
0.7466600000000000	0.1784100000000000	0.7068400000000000
0.8475600000000000	0.8083100000000000	0.7541300000000000
0.4301400000000000	1.0520200000000000	0.8295200000000000
0.2468100000000000	0.8269100000000000	0.6456200000000000
0.4413900000000000	0.6762100000000000	0.6583700000000000
0.8122200000000001	1.0015000000000001	0.3811700000000000
0.2308200000000000	0.9421700000000000	-0.1605400000000000
0.3191400000000000	0.2608200000000000	0.8069100000000000
0.0683900000000000	0.4300200000000000	1.3623700000000001
0.0080400000000000	0.6571399999999999	0.8424500000000000
-0.2329200000000000	0.1993800000000000	1.0597200000000000
1.0454600000000001	0.8128800000000000	0.1778400000000000
1.1438600000000001	0.6115900000000000	0.6583700000000000
-0.0704700000000000	1.0656000000000001	0.0007000000000000
0.8014400000000000	0.1962800000000000	0.2770500000000000
0.6473800000000000	0.4861500000000000	0.7368500000000000
0.9928600000000000	0.4700900000000000	0.5523500000000000
0.1362500000000000	0.1473700000000000	-0.0780900000000000
0.4515800000000000	0.5166700000000000	0.0620300000000000
0.5279600000000000	0.0288000000000000	0.2679600000000000
0.6224600000000000	0.4743900000000000	0.5082900000000000
0.6555600000000000	0.3450700000000000	0.3808600000000000
0.7917500000000000	0.3834200000000000	0.4991800000000000
0.5111900000000000	0.6939100000000000	0.2228700000000000
0.3447100000000000	0.8339500000000000	0.3517300000000000
0.2752900000000000	0.9447700000000000	0.3325700000000000
-0.0350200000000000	0.5428700000000000	0.0936400000000000
0.0141900000000000	0.6236400000000000	0.1783700000000000
0.5112600000000000	0.1032400000000000	0.6025000000000000
0.6291099999999999	0.0916600000000000	0.6584300000000000
0.8554000000000000	0.4765700000000000	-0.0688500000000000
0.3911400000000000	0.1800200000000000	0.4789200000000000
0.3287400000000000	0.0939200000000000	0.5356400000000000
0.4321900000000000	0.3364400000000000	0.8645000000000000

0.5959400000000000	0.8121699999999999	0.1483000000000000
0.5357400000000000	0.2670200000000000	0.3814500000000000
0.4469600000000000	0.2584700000000000	0.2802800000000000
0.6047500000000000	0.9571300000000000	0.5236300000000000
0.5719300000000000	0.9674800000000000	0.3963100000000000
0.9626900000000000	0.2052900000000000	0.2587000000000000
1.0437700000000001	0.2896800000000000	0.3149500000000000
0.1387400000000000	0.2020400000000000	0.1796000000000000
0.2670200000000000	0.2314400000000000	0.1294300000000000
0.3241500000000000	0.4138800000000000	0.7378900000000000
0.2714900000000000	0.5097200000000000	0.6689200000000000
0.6975600000000000	0.9354900000000000	0.1012600000000000
0.5808000000000000	0.9749400000000000	0.1491600000000000
0.0544200000000000	0.3833300000000000	0.8338400000000000
0.0603900000000000	0.2714000000000000	0.7609500000000000
0.8847300000000000	0.3513500000000000	0.7993800000000000
0.0011600000000000	0.0848500000000000	0.7607300000000000
0.4559800000000000	0.2720800000000000	1.0830400000000000
0.2810000000000000	0.5810000000000000	0.8569099999999999
0.2417200000000000	0.6698300000000000	0.9446400000000000
1.0059100000000001	0.9327299999999999	0.5612200000000001
0.8955300000000000	0.8712800000000001	0.6130100000000001
0.5696900000000000	0.7271100000000000	0.7341800000000001
0.6404700000000000	0.6206600000000000	0.7545800000000000
0.5873300000000000	0.6081100000000000	0.4666000000000000
0.6261500000000000	0.7366100000000000	0.4471400000000000
0.8752600000000000	0.9850200000000000	0.1069000000000000
0.8653300000000000	0.9633900000000000	0.2436400000000000
0.2055100000000000	0.9638800000000000	0.5947300000000000
0.1689700000000000	0.4847700000000000	0.9865400000000000
0.0920900000000000	0.4151300000000000	1.0462300000000000
0.8614700000000000	0.1183200000000000	0.4824500000000000
0.8729000000000000	0.2504200000000000	0.4958900000000000
0.5576100000000001	0.2370400000000000	-0.0413000000000000
0.5912900000000000	0.1339800000000000	1.0357400000000001
0.6865100000000000	0.7174400000000000	-0.1041400000000000
0.6755700000000000	0.7068500000000000	1.0331300000000001
-0.1439000000000000	-0.0678700000000000	0.7796000000000000
0.0863200000000000	-0.2540500000000000	-0.0666100000000000
0.1221300000000000	-0.1796300000000000	0.0459400000000000
-0.1792900000000000	0.0613300000000000	0.7423600000000000
0.1508400000000000	1.0032399999999999	0.4151300000000000
0.1209600000000000	1.0733500000000000	0.3059500000000000
-0.0191400000000000	0.1451400000000000	0.6472900000000000

0.8028000000000000	0.6010500000000000	-0.0275500000000000
0.4943900000000000	0.4191900000000000	0.9523100000000000
0.7615700000000000	0.4104400000000000	0.7956800000000001
0.4085300000000000	0.3865100000000000	1.1146900000000000
0.1271900000000000	1.0642900000000000	0.6027100000000000
0.7942300000000000	0.1948200000000000	0.6389000000000000
0.7583200000000000	0.2307700000000000	0.7727400000000000
0.7682800000000000	0.7795200000000000	0.7514400000000000
0.8926300000000000	0.7486800000000000	0.7853400000000000
0.4748500000000000	1.0820700000000001	0.8923300000000000
0.4894300000000000	1.0338200000000000	0.7745200000000000
0.2348000000000000	0.8663300000000000	0.7219200000000000
0.3243900000000000	0.8022800000000000	0.6456700000000000
0.4200200000000000	0.5976600000000000	0.6803399999999999
0.4798400000000000	0.6728800000000000	0.5847599999999999
0.8717800000000000	0.9553100000000000	0.4154800000000000
0.7367000000000000	0.9715000000000000	0.4070800000000000
0.2103400000000000	0.8877000000000000	-0.0992600000000000
0.3136200000000000	0.9619900000000000	-0.1582800000000000
0.3553700000000000	0.1913900000000000	0.7888500000000001
0.2497500000000000	0.2382300000000000	0.8487600000000000
0.0329500000000000	0.4527500000000000	1.4339400000000000
0.0300600000000000	0.4634000000000000	1.2987100000000000
0.0651900000000000	0.6243300000000001	0.7931700000000000
-0.0404700000000000	0.6056600000000000	0.8877000000000000
-0.1650400000000000	0.1612900000000000	1.0348900000000001
-0.2285400000000000	0.2780900000000000	1.0294700000000001
1.0768200000000001	0.8682600000000000	0.2301000000000000
0.9684900000000000	0.8452100000000000	0.1576900000000000
1.0955500000000000	0.6113000000000000	0.5926900000000001
1.1880900000000001	0.6845000000000000	0.6454900000000000
0.0105700000000000	1.0799700000000001	-0.0275800000000000
0.9036300000000000	1.0421000000000000	-0.0740800000000000
0.7835700000000000	0.1967100000000000	0.1950300000000000
0.7721300000000000	0.1226400000000000	0.3046900000000000
0.6420600000000000	0.4730600000000000	0.6536700000000000
0.5774300000000000	0.4567700000000000	0.7680000000000000
0.9089600000000000	0.4692900000000000	0.5548400000000000
1.0150100000000000	0.4364700000000000	0.6224900000000000
0.1636500000000000	0.0735400000000000	-0.1031000000000000
0.1611000000000000	0.1626600000000000	0.0040200000000000
0.3942400000000000	0.5501400000000000	0.0121000000000000
0.4620300000000000	0.5605900000000000	0.1292000000000000
0.5093400000000000	0.1021400000000000	0.3123400000000000

0.4573200000000000	-0.0145900000000000	0.2696800000000000
0.5305800000000001	0.7070700000000000	0.3059600000000000
0.7146900000000000	0.2887200000000000	0.3541900000000000
0.6946800000000000	0.4059900000000000	0.4681900000000000

The structure of $CO_2 + H_2O_2 + OH^-$ with 49 H_2O molecules employed in the AIMD simulations.

O54H101C1
1.0000000000000000

12.0000000000000000	0.0000000000000000	0.0000000000000000
0.0000000000000007	12.0000000000000000	0.0000000000000000
0.0000000000000007	0.0000000000000007	12.0000000000000000

O H C
54 101 1
Direct

-0.0438900000000000	1.1513500000000001	0.5897600000000000
0.2850900000000000	0.7708900000000000	0.1203300000000000
0.7921700000000000	0.1558900000000000	0.8289200000000000
0.2207600000000000	0.1746800000000000	0.5642500000000000
0.3092300000000000	0.4493500000000000	0.4918600000000000
0.7040500000000000	0.2316100000000000	0.3525900000000000
0.3184000000000000	-0.0099900000000000	0.0603600000000000
0.6773100000000000	0.1487400000000000	0.1381000000000000
0.3175700000000000	0.2414900000000000	0.3641100000000000
0.5798600000000000	0.8345000000000000	0.9923400000000000
0.2012200000000000	0.5199900000000000	0.2843600000000000
0.5556300000000000	0.1453800000000000	0.7963600000000000
0.3069200000000000	0.4562600000000000	0.8565500000000000
0.0087800000000000	0.7654900000000000	0.4851800000000000
0.6530000000000000	0.8589700000000000	0.5671100000000000
0.8233100000000000	0.8513400000000000	0.4051400000000000
0.7593400000000000	0.7879200000000000	0.1964700000000000
0.2708700000000000	0.8910500000000000	0.5211300000000000
0.0774100000000000	0.9318500000000000	0.6116300000000000
0.5194000000000000	0.5620400000000000	0.5176500000000001
0.4827300000000000	-0.0441900000000000	0.4208900000000000
0.8366600000000000	0.4142700000000000	0.3811500000000000
0.2501000000000000	0.3916600000000000	0.0704800000000000
0.4411800000000000	0.3152700000000000	0.1395600000000000
0.1604600000000000	0.8140100000000000	0.3371400000000000
0.9962600000000000	0.4251900000000000	0.2544300000000000
0.1718600000000000	0.4170200000000000	0.6616400000000000

0.9433200000000000	0.5385000000000000	0.0521000000000000
0.5184400000000000	0.1802700000000000	0.4597000000000000
0.6823700000000000	0.6642800000000000	0.8871300000000000
0.8776300000000000	0.1140000000000000	0.0434200000000000
1.0204000000000000	0.7088900000000000	0.9481300000000000
0.5697500000000000	0.6820600000000000	0.6893600000000000
0.3767700000000000	0.8119000000000000	0.7236399999999999
0.8633999999999999	0.7338000000000000	0.7870800000000000
0.4857000000000000	0.0531500000000000	0.2168000000000000
0.4335000000000000	0.9711100000000000	0.8693600000000000
0.5276500000000000	0.3836900000000000	0.8122700000000000
0.9018300000000000	0.8834900000000000	0.0592400000000000
0.0196800000000000	0.2599300000000000	0.1252000000000000
0.0482700000000000	0.9870600000000000	0.2547500000000000
0.8494400000000000	0.0762100000000000	0.3941000000000000
0.7506400000000000	0.9631500000000000	0.7482600000000000
0.2685300000000000	0.6796700000000000	0.8797400000000000
0.7155100000000000	0.5619800000000000	0.0695600000000000
0.4755400000000000	0.6747400000000000	0.1623600000000000
0.9543700000000001	0.5907400000000000	0.6280300000000000
0.8903600000000000	0.3762000000000000	0.6107500000000000
0.1804400000000000	0.1358100000000000	0.1457100000000000
0.6361700000000000	0.3308700000000000	0.0143800000000000
0.0248300000000000	0.3855600000000000	0.8558500000000000
0.6289300000000000	0.5466800000000001	0.2784200000000000
0.8323100000000000	0.4080300000000000	0.8391999999999999
0.7061900000000000	0.5733100000000000	0.3682700000000000
-0.0726100000000000	0.1272900000000000	0.5125500000000000
-0.0217700000000000	1.0779799999999999	0.6257300000000000
0.2482200000000000	0.7658600000000000	0.1936800000000000
0.3579400000000000	0.7312100000000000	0.1366300000000000
0.7901200000000000	0.0822900000000000	0.7874500000000000
0.8034400000000000	0.2208500000000000	0.7837499999999999
0.8654400000000000	0.5529600000000000	0.0683400000000000
0.2093000000000000	0.2385700000000000	0.6133600000000000
0.1486100000000000	0.1478100000000000	0.5493000000000000
0.5801300000000000	0.1947600000000000	0.4067000000000000
0.3202600000000000	0.3778900000000000	0.4512800000000000
0.3852400000000000	0.4774100000000000	0.5101800000000000
0.7600000000000000	0.1765500000000000	0.3795900000000000
0.6990000000000000	0.2137200000000000	0.2712400000000000
0.2743500000000000	0.0552600000000000	0.0786100000000000
0.2851600000000000	0.9213600000000000	0.0943000000000000
0.6644600000000001	0.2026700000000000	0.0776000000000000

0.7554600000000000	0.1262800000000000	0.1190400000000000
0.3921500000000000	0.2056400000000000	0.3714100000000000
0.2695300000000000	0.2101600000000000	0.4263600000000000
1.0173700000000001	0.3576000000000000	0.2058400000000000
0.0691900000000000	0.4588400000000000	0.2834800000000000
0.6128100000000000	0.8966100000000000	1.0302700000000000
0.5322500000000000	0.8775200000000000	0.9381800000000000
0.1909600000000000	0.5981700000000000	0.2736600000000000
0.2418000000000000	0.5094100000000000	0.3582100000000000
0.7590500000000000	0.6758500000000000	0.8517200000000000
0.2238300000000000	0.4271700000000000	0.5992200000000000
0.6342800000000000	0.1390000000000000	0.8166200000000000
0.2918600000000000	0.5412000000000000	0.8657600000000000
0.2575600000000000	0.4413100000000000	0.7908900000000000
0.9386800000000000	0.7960800000000000	0.4540300000000000
0.0505300000000000	0.8762300000000000	0.5549100000000000
0.7664600000000000	0.8438200000000000	0.4631700000000000
0.5889600000000000	0.8694800000000000	0.5143900000000000
0.7678199999999999	0.5137800000000000	0.3754700000000000
0.7955000000000000	0.8240499999999999	0.3316700000000000
0.7516800000000000	0.7094800000000000	0.1806300000000000
0.8256800000000000	0.8108400000000000	0.1529500000000000
0.3357700000000000	-0.0612900000000000	0.5163700000000000
0.5406800000000000	0.6109599999999999	0.5842300000000000
0.4878200000000000	-0.0451200000000000	0.3423100000000000
0.5103000000000000	0.0302100000000000	0.4415200000000000
0.7758300000000000	0.3544300000000000	0.3614100000000000
0.9092800000000000	0.4127000000000000	0.3395000000000000
0.2203600000000000	0.4563300000000000	0.1122200000000000
0.2629100000000000	0.4139900000000000	0.9926500000000000
0.0779000000000000	0.8894600000000000	0.6836200000000000
0.3783700000000000	0.3623200000000000	0.1183200000000000
0.4156500000000000	0.2372000000000000	0.1510900000000000
0.2115600000000000	0.9179100000000000	0.5806300000000000
0.0690600000000000	0.7661300000000000	0.4225400000000000
0.2104100000000000	0.8424500000000000	0.3943800000000000
0.1166800000000000	0.4781800000000000	0.6465100000000000
0.9633600000000000	0.5081500000000000	0.1253400000000000
0.5358000000000001	0.2217400000000000	0.5275600000000000
0.6562800000000000	0.7382700000000000	0.9128700000000000
0.5452000000000000	0.2277500000000000	0.7928700000000000
0.3263600000000000	0.8344800000000000	0.6603000000000000
0.8649900000000000	0.1493700000000000	0.9723900000000000
0.9268000000000000	0.1715000000000000	0.0817900000000000

0.9916800000000000	0.6349399999999999	0.9868000000000000
0.0949400000000000	0.6835200000000000	0.9243500000000000
0.6181700000000000	0.7908700000000000	0.6092000000000000
0.6177300000000000	0.6575100000000000	0.7566300000000000
0.5009700000000000	0.7241400000000000	0.7176800000000000
0.3898100000000000	0.8762700000000000	0.7805000000000000
0.8802500000000000	0.6819900000000000	0.7257900000000000
0.9218600000000000	0.7250300000000000	0.8462600000000000
0.5621400000000000	0.0716500000000000	0.1891300000000000
0.4394500000000000	0.0157000000000000	0.1552300000000000
0.4777100000000000	0.0421900000000000	0.8516300000000000
0.3782000000000000	0.9782200000000000	0.9315800000000000
0.4472900000000000	0.4167000000000000	0.8279600000000000
0.5382300000000000	0.4086900000000000	0.7333700000000000
0.8952400000000000	-0.0357000000000000	0.0557100000000000
0.9526200000000000	0.8377400000000000	0.0135100000000000
0.1074300000000000	0.2012300000000000	0.1418900000000000
0.0367200000000000	0.2827900000000000	0.0490200000000000
0.1248800000000000	0.8883900000000000	0.3137500000000000
-0.0079400000000000	0.9526100000000000	0.2124400000000000
0.8329600000000000	0.9961100000000001	0.4101800000000000
0.9238800000000000	1.0754300000000001	0.3503800000000000
0.8022700000000000	0.8989600000000000	0.7617400000000000
0.6972300000000000	0.9333300000000000	0.6937100000000000
0.2712600000000000	0.6977300000000000	0.9564900000000000
0.3054400000000000	0.7353800000000000	0.8308700000000000
0.7022000000000000	0.5878000000000000	0.9899600000000000
0.7027700000000000	0.4776300000000000	0.0648500000000000
0.5202800000000000	0.6340700000000000	0.2196400000000000
0.5279400000000000	0.6963400000000000	0.1015100000000000
0.9838300000000000	0.6271000000000000	0.5602200000000001
0.9335100000000000	0.5137900000000000	0.6059700000000000
0.9231800000000000	0.3011900000000000	0.6044500000000000
-0.1282400000000000	0.3880900000000000	0.5288100000000000
0.0912300000000000	0.0466400000000000	0.2076800000000000
0.2331500000000000	0.1777100000000000	0.1957100000000000
0.6172200000000000	0.3503500000000000	0.9383300000000000
0.5604300000000000	0.3349900000000000	0.0569500000000000
0.6835000000000000	0.5458000000000000	0.2181200000000000
0.5910000000000000	0.5658600000000000	0.4683300000000000
-0.0725300000000000	0.3899400000000000	0.8571400000000000
